# Feasibility and Safety of Field-Based Physical Fitness Tests: A Systematic Review

**DOI:** 10.1186/s40798-024-00799-1

**Published:** 2025-01-24

**Authors:** Carolina Cruz-León, Pablo Expósito-Carrillo, Sandra Sánchez-Parente, José Jiménez-Iglesias, Milkana Borges-Cosic, Magdalena Cuenca-Garcia, José Castro-Piñero

**Affiliations:** 1 GALENO Research Group, Department of Physical Education, Faculty of Education Sciences, University of Cadiz, Avenida República Saharaui s/n, 11519 Puerto Real, Cádiz, Spain; 2 Instituto de Investigación e Innovación Biomédica de Cádiz (INiBICA), Cádiz, Spain; 3 Sport Science Department Cadiz C.F., Cadiz C.F., Cadiz, Spain

**Keywords:** Feasibility, Safety, Cardiorespiratory fitness, Muscular strength, Motor fitness, Fitness test

## Abstract

**Background:**

While there is evidence on the validity and reliability of field-based physical fitness tests in children, adolescents and adults, there is limited evidence to provide feasibility and safety data on the application and performance of the existing field-based physical fitness tests.

**Objectives:**

(i) To examine the feasibility and safety of existing field-based physical fitness tests used in people of all ages and (ii) to establish a comprehensive view of criterion-related validity, reliability, feasibility and safety based on scientific evidence for the existing field-based physical fitness tests in adults.

**Methods:**

The search was conducted through the electronic databases MEDLINE (via PubMed) and Web of Science (all databases) for published studies from inception to 31 January 2023. This systematic review was developed according to Preferred Reporting Items for Systematic Reviews and Meta-Analysis (PRISMA) guidelines. Studies were classified as very low quality, low quality or high quality, based on the criteria of appropriate number of participants, appropriate description of the study population, and appropriate number of items reported to assess feasibility/safety. Three evidence levels were constructed (strong, moderate and limited or inconclusive evidence) according to the number of studies and the consistency of the findings.

**Results:**

We identified a total of 19 (14 of high quality) and 13 (11 high quality) original studies examining the feasibility and safety of field-based physical fitness tests, respectively. Strong evidence indicated that (a) the 2-km walk and 20-m shuttle run tests were feasible in adults and, children and adolescents, respectively; (b) the handgrip strength test was feasible in children and adolescents; and (c) the standing long jump test was feasible in children and adolescents. Only the 2-km walk test has shown strong evidence on safety in adults. Finally, combining the levels of evidence of criterion-related validity, reliability, feasibility and safety in adults, all the field-based physical fitness tests show limited evidence.

**Conclusion:**

There is a need for more studies and consensus to establish homogeneous methodological criteria to assess the feasibility and safety of field-based fitness tests. The combined evidence on criterion-related validity, reliability, feasibility and safety of field-based tests was found to be limited in adults.

*PROSPERO reference number* CRD42022298276.

**Supplementary Information:**

The online version contains supplementary material available at 10.1186/s40798-024-00799-1.

## Key Points

- There is little scientific evidence about the feasibility and safety of field-based physical fitness tests at all ages, especially in older adults.
- There is no consensus on the assessment of feasibility and safety of field-based physical fitness tests for people of all ages.
- This review provides an evidence-based proposal of the most commonly used items to assess feasibility and safety of field-based physical fitness tests for people of all ages.
- This study provides complementary information to our previous studies about the criterion-related validity and reliability of field-based physical fitness tests in adults conducted under the umbrella of the ADULT-FIT project. The combined evidence on criterion-related validity, reliability, feasibility and safety of field-based tests is limited in adults due to the lack of evidence on feasibility and safety.

**Supplementary Information:**

The online version contains supplementary material available at 10.1186/s40798-024-00799-1.

## Introduction

There is considerable evidence of the positive effects of physical fitness on health at all ages. Appropriate levels of physical fitness are associated with a healthy cardiometabolic profile [1], quality of life (QoL) [2], improvements in bone health [3, 4], positive effect on mental health [5] and better cognitive function [6] in children and adolescents. Moreover, cardiorespiratory fitness and muscular strength are inversely associated with cardiovascular and metabolic diseases [7], obesity [8], diabetes [9], different types of cancer [10, 11], cognitive impairment [12], mental health [13], bone health [14] and a predictor of all-cause of mortality and cardiovascular disease-related mortality [15, 16] in adults and older adults. Regarding motor fitness, evidence supports that slower gait speed, worse agility and impaired balance are significantly associated with an increased risk of adverse health outcomes, such as falls and fear of falling, mobility disability, CVD risk or all-cause mortality in adult and older adults [17]. Based on this evidence, the assessment of physical fitness at all ages is relevant from a public health point of view.

Physical fitness can be objectively and accurately assessed through laboratory tests [18], i.e. always in a controlled and safe environment. However, most of these tests are expensive, not easily transportable, the evaluation time is too long and does not permit the assessment of a large number of people at the same time. Properly conducted field-based physical fitness tests provide a simple, low-cost alternative, are easy to implement and are time-efficient, feasible, safe and allows evaluation of a large number of people simultaneously.

A field-based physical fitness test that assesses health-related physical fitness must meet a number of fundamental requirements [18], that is, it must be (i) related to the present and future health of the individual, (ii) valid, (iii) reliable, (iv) feasible and (v) safe to perform, both in clinical settings and in epidemiological studies.

Feasibility and safety are two important components when assessing physical fitness. The feasibility refers to the evaluation of those components that will give the necessary justification for successful testing, taking into account factors such as the acceptance of participants, time needed to implement and execute, optimal infrastructure, among others [19]. On the other hand, safety can be considered as the component that assesses the health of participants before, during and after the test. A test is considered safe when it does not cause physical complications to participants [20].

While there is evidence on the validity and reliability of field-based physical fitness tests in children, adolescents [21, 22] and adults [23, 24]; there is limited evidence to provide feasibility and safety data on the application and performance for the existing field-based physical fitness tests in children, adolescents, adults and older adults.

On the other hand, it would be desirable to have a comprehensive view of criterion-related validity, reliability, feasibility and safety based on scientific evidence for the existing field-based physical fitness tests in adults. This will make it possible to create the basis of a proposal for a valid, reliable, feasible, safe and responsive health-related field-based physical fitness tests battery in adults, this being the objective of our project called “ADULT-FIT project”.

Therefore, the main aim of the present systematic review was to study the feasibility and safety of the existing field-based physical fitness tests in people of all ages. In addition, the second aim was to establish a comprehensive view of criterion-related validity, reliability, feasibility and safety based on scientific evidence of the existing field-based physical fitness tests in adults.

## Methods

### Protocol and Registration

This systematic review was developed according to the Preferred Reporting Items for Systematic Reviews and Meta-Analysis (PRISMA) guidelines [25]. The review protocol was registered in the International Prospective Register of Systematic Reviews (PROSPERO reference number, CRD42022298276) [26].

### Data Sources and Search Strategy

A systematic search was performed of the electronic databases MEDLINE (via PubMed) and Web of Science (all databases) from database inception to 31 January 2023. The search terms used were those related to the following topics: (i) feasibility: feasibility, viability; (ii) safety: safety; and (iii) physical fitness components: physical fitness, muscular strength, range of motion, articular, postural balance, physical endurance, cardiorespiratory fitness, cardiovascular fitness, aerobic fitness, aerobic capacity, maximal oxygen consumption, VO2max, motor fitness, running speed and agility. The three search topics were combined with the Boolean operators ‘AND’ (inter topics) and ‘OR’ (intra topics). When using PubMed, we included Medical Subject Heading (MeSH) terms to enhance the power of the search. The same search strategy and combination of terms was repeated in Web of Science but without using MeSH terms or their equivalent as a similar option does not exist for this database (see Supplementary Material 1).

### Eligibility Criteria

The inclusion criteria for this systematic review were: (i) ages: all ages; (ii) participants: the study population was based on a generally healthy population, who did not present any injury, physical and/or mental disabilities, irrespective of body mass index (BMI), diabetes or other cardiovascular risks (i.e., hypertension, hypercholesterolemia, lipid profiles, glucose levels, insulin sensitivity). In the course of this review, we found that some studies sampled healthy subjects and those with pathologies together. In those cases, we assessed whether these studies performed stratified analyses by groups, separating the population with pathologies from the rest; if so, we included it in the study; (iii) study design: original studies or systematic reviews/meta-analyses; (iv) language: articles were only published in English or Spanish; and (v) topic criterion: original studies analysing feasibility and safety in field-based physical fitness tests. We did not include studies that examined the feasibility and safety of field-based physical fitness tests designed for use in exclusive clinical settings.

### Study Selection

The study selection process was carried out in three stages. Firstly, records were identified via the PubMed and Web of Science databases and imported into the EndNote software (Desktop version 20.1). Duplicate files were then removed, initially automatically by the software and secondarily by visual checking. Secondly, the titles and abstracts of the search results were checked for eligibility. Finally, those full-text reports that were apparently eligible were read for final inclusion or exclusion in the review.

The results of all stages were compared between two researchers (C.C.L and P.E.C). If no consensus was reached between the two investigators (< 5%), a third investigator (J.C.P) made the final decision. Furthermore, the literature search was complemented by manual review of the reference lists obtained from the selected studies. The authors were not blinded to the selected articles, as the reviewers who performed the quality assessment were familiar with the literature.

### Data Extraction

Two researchers (C.C.L. and P.E.C.) independently extracted the following information from each eligible original study according to the standardized form: (i) the author’s name, (ii) sex and number of participants, (iii) age of participants, (iv) field-based physical fitness test, (v) statistical methods (vi) main outcome and (vii) conclusions. Any disagreement in the extracted data was discussed among the researchers until a consensus was reached.

### Risk of Bias Assessment

Risk of bias was assessed by two independent investigators (C.C.L and P.E.C). They obtained 94% agreement on the quality assessment before consensus, and 100% agreement after resolving discrepancies in a consensus meeting, with a third investigator (J.C.P). Risks of bias of the original studies were determined, modifying the original quality assessment checklist developed by Castro-Piñero et al. [22], based on three criteria: (i) appropriate number of participants, (ii) appropriate description of the study population and (iii) appropriate number of items reported, to assess feasibility/safety (see Supplementary Tables S1 and S2). Each criterion was scored from 0 to 2 (where 2 is the best score). The scores for each criterion were summarized (0–6) for all studies. Studies were classified as very low quality (score ≤ 2), low quality (score 3–4) or high quality (score ≥ 5).

### Level of Evidence

To formulate a final conclusion on the feasibility and safety of the field-based physical fitness test, we determined three levels of evidence [22]: (i) strong evidence: consistent findings in three or more high-quality studies; (ii) moderate evidence: consistent findings in two high quality studies; and (iii) limited or inconclusive evidence: consistent findings in multiple low-quality studies, inconsistent results found in multiple high-quality studies, or results based on one single study (see Tables 1 and 2).

**Table 1:**
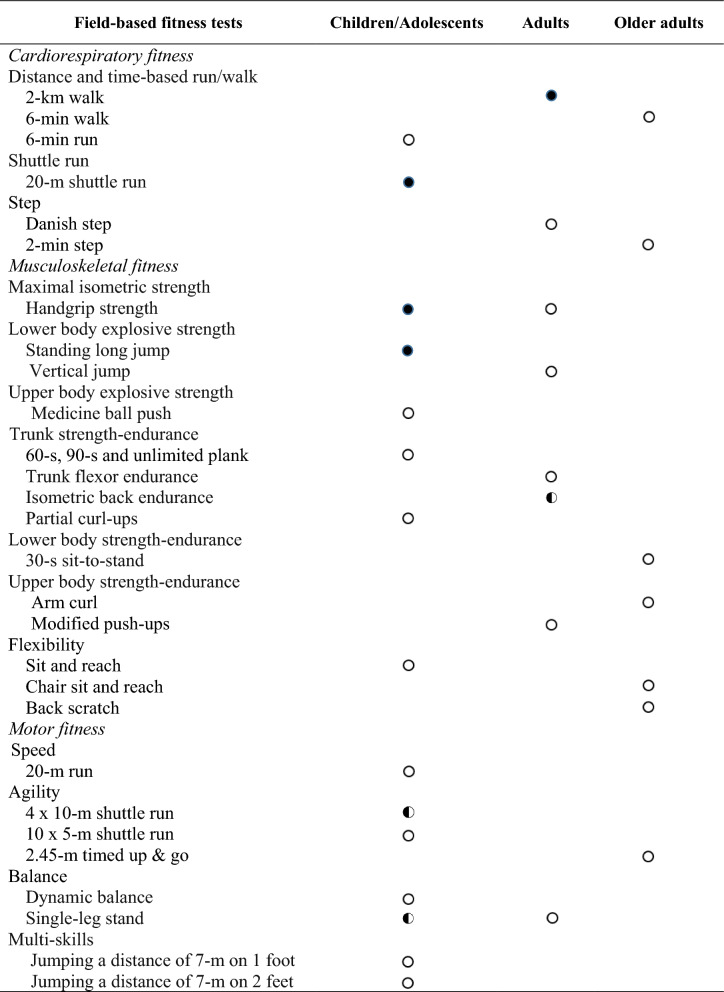
Levels of evidence of feasibility in field-based fitness tests by age group

Indicates strong evidence:

Indicates moderate evidence:

Indicates limited evidence:

**Table 2:**
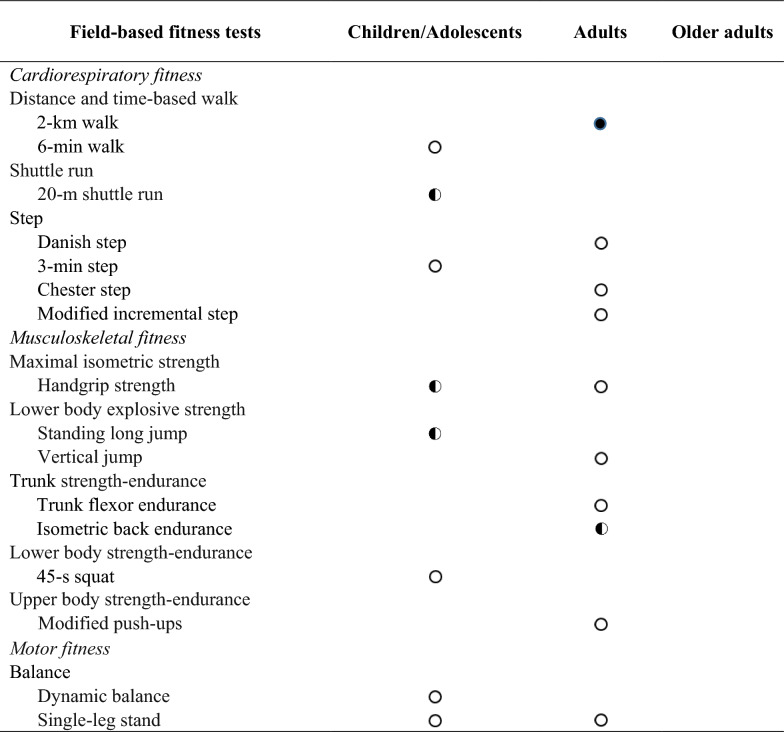
Levels of evidence of safety in field-based fitness tests by age group

Indicates strong evidence:

Indicates moderate evidence:

Indicates limited evidence:

Regarding the levels of evidence on the comprehensive view of criterion-related validity, reliability, feasibility and safety for each field-based physical fitness test, based on evidence from previous reviews [23, 24] and this one, we determined three levels of evidence: (i) strong evidence: when the field-based physical fitness test showed strong level of evidence in criterion-related validity, reliability, feasibility and safety; (ii) moderate evidence: when the field-based physical fitness test showed moderate evidence in all studied qualities (i. e., criterion-related validity, reliability, feasibility and safety), or at least moderate or strong evidence in some of them; and (iii) limited evidence: when the field-based physical fitness test showed neither moderate nor strong evidence in some of the studied qualities (i.e., criterion-related validity, reliability, feasibility or safety) (see Tables 3 and 4).

**Table 3:**
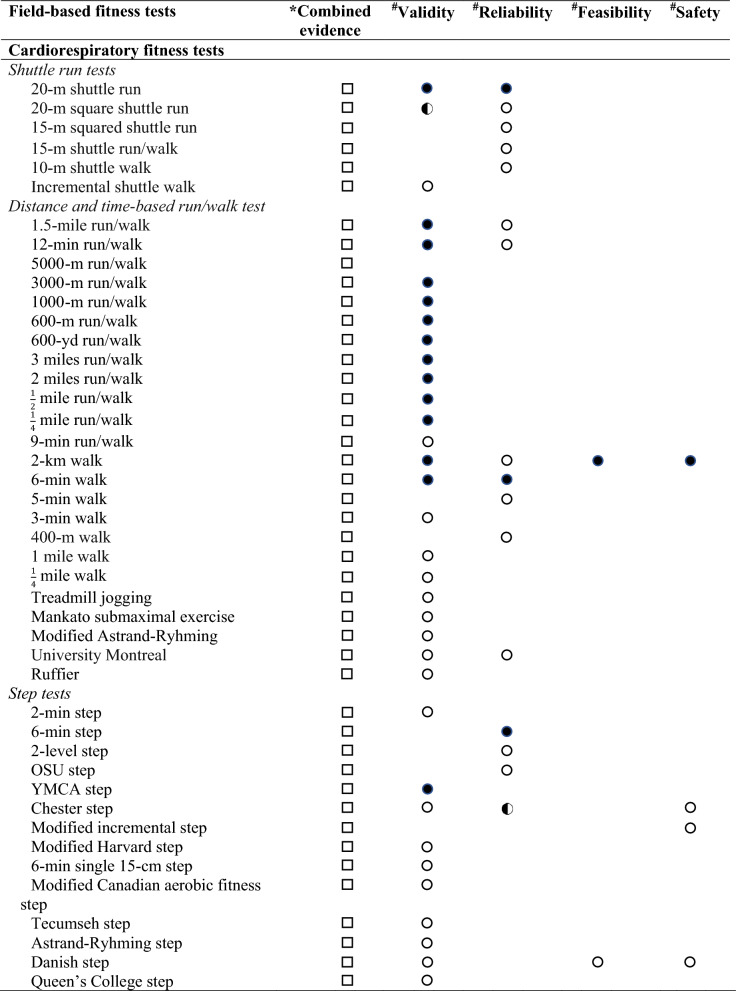
Overview of evidence of criterion-related validity [23], reliability [24], feasibility and safety of field-based cardiorespiratory fitness tests in adults

*COMBINED evidence. Three levels were set: ( ) strong evidence: when the field-based physical fitness test showed strong level of evidence in validity, reliability, feasibility and safety; ( ) moderate evidence: when the field-based physical fitness test showed moderate evidence in all studied qualities (i.e., validity, reliability, feasibility and safety), or at least moderate or strong evidence in some of them; and ( ) limited evidence: when the field-based physical fitness test showed neither moderate or strong evidence in some of the studied qualities (i.e., validity, reliability, feasibility or safety)

^**#**^INDIVIDUAL evidence. Three levels were set: ( ) strong evidence: consistent findings in three or more high-quality studies; ( ) moderate evidence: consistent findings in two high quality studies; and ( ) limited or inconclusive evidence: consistent findings in multiple low-quality studies, inconsistent results found in multiple high-quality studies, or results based on one single studyand

**Table 4:**
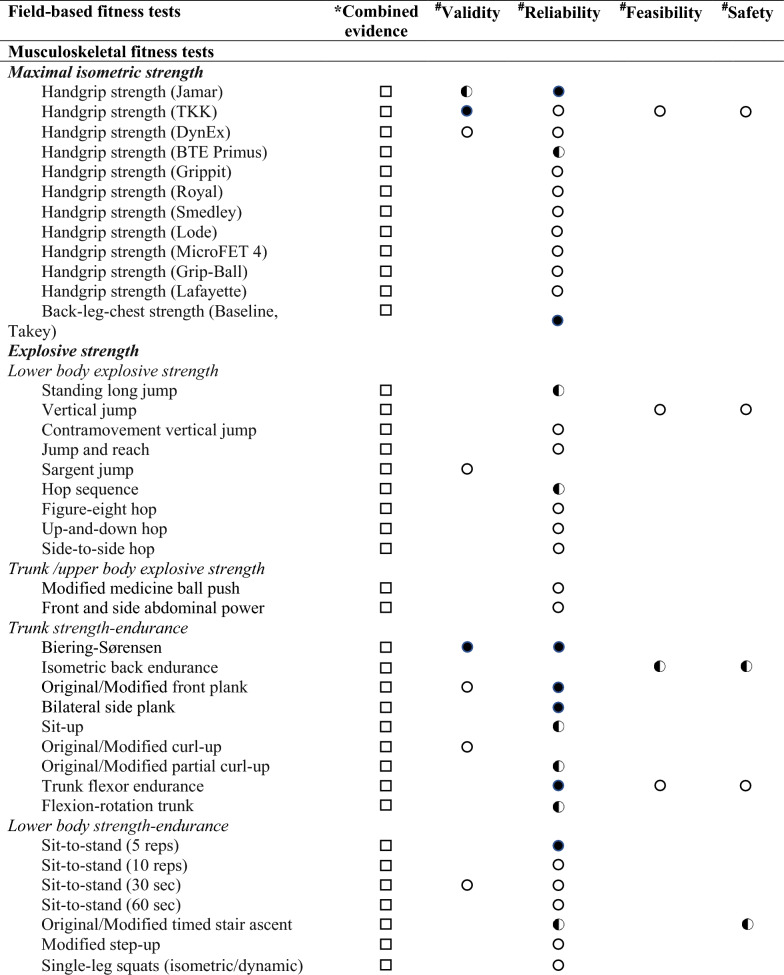

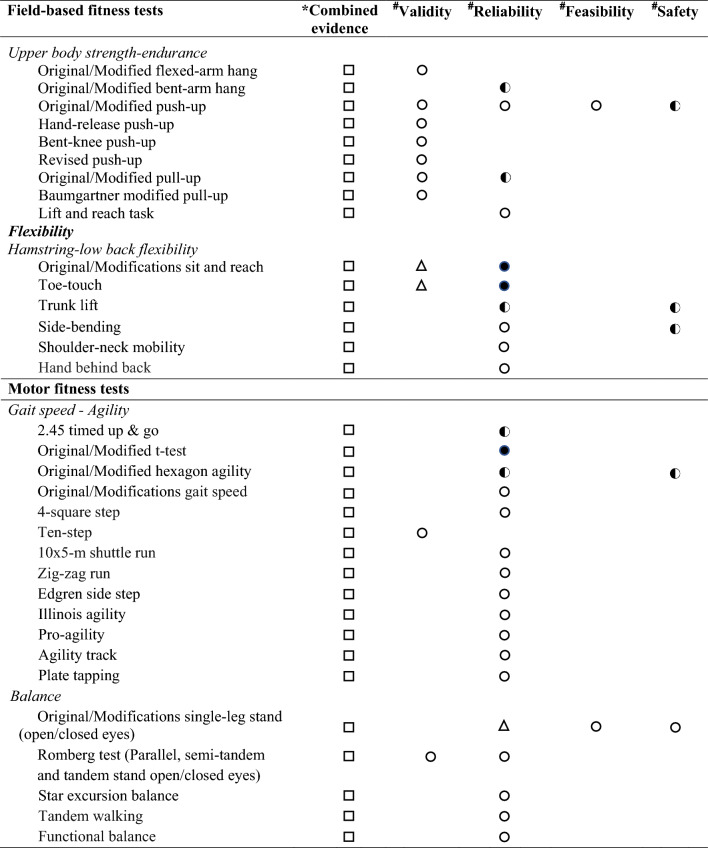
Overview of evidence of criterion-related validity [23], reliability [24], feasibility and safety of field-based musculoskeletal and motor fitness tests in adults

*COMBINED evidence. Three levels were set: ( ) strong evidence: when the field-based physical fitness test showed strong level of evidence in validity, reliability, feasibility and safety; ( ) moderate evidence: when the field-based physical fitness test showed moderate evidence in all studied qualities (i.e., validity, reliability, feasibility and safety), or at least moderate or strong evidence in some of them; and ( ) limited evidence: when the field-based physical fitness test showed neither moderate or strong evidence in some of the studied qualities (i.e., validity, reliability, feasibility or safety)

^#^INDIVIDUAL evidence. Three levels were set: ( ) strong evidence: consistent findings in three or more high-quality studies; ( ) moderate evidence: consistent findings in two high quality studies; and ( ) limited or inconclusive evidence: consistent findings in multiple low-quality studies, inconsistent results found in multiple high-quality studies, or results based on one single study. (△) Strong evidence that the test is not validity, reliability, feasibility or safety

## Results

**Fig. 1:**
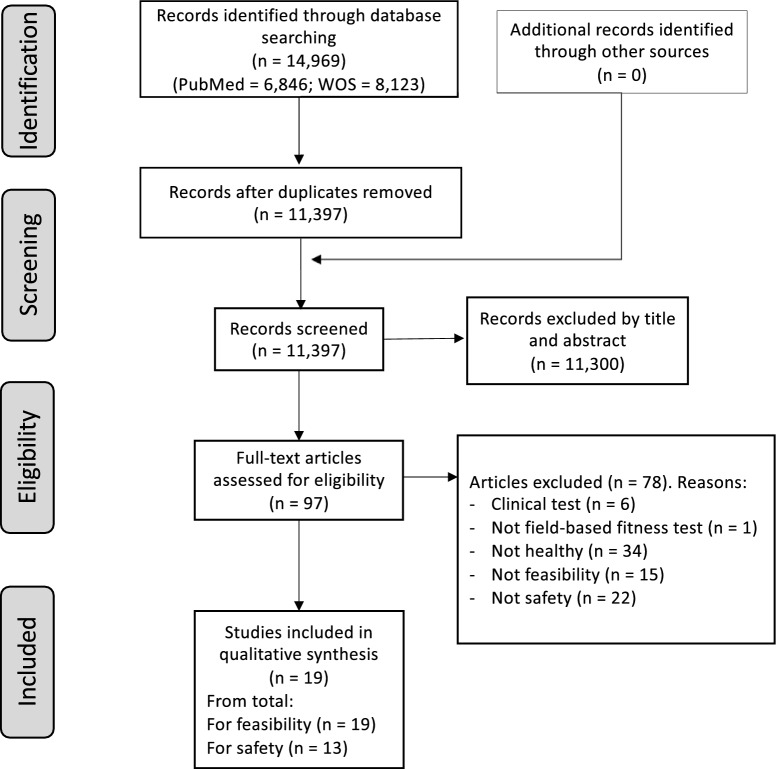
PRISMA flowchart showing the selection of original studies

The literature search identified 14,969 studies (see the PRISMA flowchart in Fig. 1). After the removal of duplicate references (3572 studies), and the screening of titles and abstracts (11,300 studies), we excluded 14,872 studies. A total of 97 full-text studies were assessed for eligibility, and 78 were excluded due to reasons indicated in Fig. 1. Finally, a total of 19 original studies addressed the feasibility and 13 of them also studied the safety of field-based physical fitness tests.

One study was classified as “very low quality” (score ≤ 2) for feasibility [27]. Four studies were classified as “low quality” (score 3–4) for feasibility [28–31] and 2 for safety [30, 32]. Fourteen studies were classified as “high quality” (score ≥ 5) for feasibility and 11 for safety (see Supplementary Tables S3 and S4).

The sample size in the feasibility studies was 4186 participants, of which 2124 were children and adolescents, 1857 were adults and 205 were older adults (see Supplementary Table S5). From the 19 original studies, 14 specified the sex of participants with a proportion of females of 26% (n = 991) for children and adolescents, and 27.4% (n = 1037) for adults. None of the studies specified sex in older adults.

The sample size in the safety studies was 2665 participants, of which 808 were children and adolescents and 1857 were adults (see Supplementary Table S6). From the 13 original studies, 10 specified the sex of participants with a proportion of females of 13.4% (n = 334) for children and adolescents, and 41.6% (n = 1037) for adults. None of the studies specified sex in older adults.

The feasibility and safety of the field-based physical fitness tests were measured through questionnaires. Fourteen items were identified to assess feasibility (reported by evaluators and participants) and 11 items were observed to assess safety of the field-based physical fitness tests (reported by evaluators and by participants). Most of the studies used the frequency in number or percentages to determine feasibility and safety. Heart rate (HR) and scales data are presented as mean and standard deviation.

Supplementary Table S7 shows a description (i.e., fitness component, equipment used, procedures and scoring) for each one of the field-based physical fitness tests included in the systematic review.

### Feasibility

A synthesized version of Supplementary Table S5 is presented as Supplementary Table S9 for easy reading and condensation of the results.

#### Cardiorespiratory Fitness

##### Distance and Time-Based Run/Walk Tests

Five high-quality studies [20, 33–36] have analysed the feasibility of the distance run/walk test (see Supplementary Tables S5 and S8). Three studies [20, 34, 35] showed that the 2-km walk test was feasible (~ 20% of the participants declined to perform the test, the time to perform the test was ~ 15 to 18 min, two or three evaluators assessed the test and 81% of the participants found the test “easy”) in adults. One study [33] showed that the 6-min walk test was feasible (100% of the participants performed the test) in older adults. One study [36] reported that the 6-min run test was feasible (100% of the participants performed the test and they understood test instructions, and the test was considered easy to administer) in children.

Only one low-quality study [29] found that the 6-min walk test was feasible (100% of the participants performed the test and the time to perform it was 6 min) in adults.

##### 20-m Shuttle Run Test

Four high-quality studies [32, 37–39] analysed the feasibility of the 20-m shuttle run test in children (see Supplementary Tables S5 and S8). All of them showed that this test was feasible (8 to 10 min to perform the test, a minimum of 2 evaluators assessed the test [37, 38]; groups of 4 to 8 participants, 4 to 5 participants if they are 3 years old [37]; groups of 4 to 8 participants of the same age, 100% of the participants understood test instructions and the test was easy to administer [38]; 96% of the participants performed the test, and the time to perform it was 6.7 ± 3 min for males and 5.3 ± 2 min for females [32]; 5 min tthe test because of lack of motivationo prepare and 10 min to perform the test approximately, 20 participants per group, 1 physical education teacher as evaluator, 99.2% of participants wore appropriate sport clothes, 100% of participants understood test instructions, 0.8% declined to perform , 100% of facilities were appropriate and the test was easy to administer [39]).

Only one low-quality study [31] compared the original 20-m shuttle run test versus the music version of 20-m shuttle run test through a self-reported questionnaire. Both tests were feasible, but the version with music showed a higher preference (89.6%) and enjoyment (94%), and less fatigue perception (56.4%), while the percentage of participants who achieved the maximum effort was similar in both tests (72.4%).

##### Step Tests

Two high-quality studies [33, 40] analysed the feasibility of the step tests (see Supplementary Tables S5 and S8). The Danish step test [40] and the 2-min step test [33], used to assess cardiorespiratory fitness, were feasible (99% and 100% of participants performed the test and the time to perform was 6 and 2 min, respectively) in adults and older adults, respectively.

Three low-quality studies [28–30] analysed the feasibility of the step tests. The 3-min step test [28] was feasible (100% of the participants found the tests to be “low effort” and the test was considered easy to administer) in children and adolescents. The Chester step and modified incremental step tests [29] were considered feasible (10 and 12 min to perform the tests, respectively, and 100% of the participants performed the tests) in adults. Moreover, the 6-min step test [30] was found to be feasible (6 min to perform the test and 100% of the participants performed the test) in adults.

#### Muscular Strength

##### Maximal Isometric Strength

Five high-quality studies [20, 32, 38, 39, 41] analysed the feasibility of hand maximal isometric strength, using the handgrip strength test (see Supplementary Tables S5 and S8). Four studies [32, 38, 39, 41] showed that the handgrip strength test that assessed maximal isometric muscular strength was feasible (100% of acceptance for the test [32, 41]; 100% of the participants performed the test [32]; 8 to 10 min to perform the test, 8 participants per group, 100% of the participants understood the test instructions correctly and the test was considered easy to administer [38]; ~ 3 min of preparation, ~ 90 s to perform the test by participant, 20 participants per group, 1 physical education teacher as evaluator, 99.2% of participants wore appropriate sport clothes, 98.5% of participants understood test instructions, no one declined to perform the test, 100% of facilities were appropriate and the test was considered easy to administer [39] in children and adolescents. One study [20] reported that the handgrip strength was feasible (time to prepare and perform the test on a 5 points scale: mean score = 4.3–4.7 (excellent) and 95% of the participants performed the test) in adults.

##### Explosive Strength

Six high-quality studies [20, 32, 36, 38, 39, 42] assessed the feasibility of lower body explosive strength tests (see Supplementary Tables S5 and S8). These studies reported that the standing long jump test to assess lower body explosive strength, was feasible (100% of the participants performed the test [32, 36, 42]; 100% of the participants understood test instructions and the test was considered easy to administer [36]; 8 to 10 min to perform the test, maximum 8 participants per group, 100% of the participants gave a positive answer about “understanding the test instructions” and the test was considered difficult to administer (several trials were required) [38]; ~ 3 min of preparation, 50 s to perform the test by participant, 20 participants per group, 1 physical education teacher as evaluator, 99% of participants wore appropriate sport clothes and understood the test instructions, 0.8% of participants declined to perform the test because of lack of motivation, 100% of facilities were appropriate and the test was considered easy to administer [39]; 100% of the participants performed the test and understood the test instructions, 76% of participants wore appropriate sport clothes, the test was considered easy to administer and the space to administer the test was appropriate [42] in children and adolescents. Finally, one study [20] showed that the vertical jump test was considered feasible (time to prepare and perform the test on a 5 points scale: mean score = 5.0 (excellent) and 95% of the participants performed the test) in adults.

One high-quality study [36] assessed the feasibility of the upper body explosive strength test (see Supplementary Tables S5 and S8). This study reported that the medicine ball push test was feasible in children (100% of the participants performed the test, 100% of participants understood the test instructions and the test was considered easy to administer).

##### Strength-Endurance

Three high-quality studies [20, 43, 44] assessed the feasibility of trunk strength-endurance tests (see Supplementary Tables S5 and S8). One study [43] analyzed the feasibility of the partial curl-ups, 60-s, 90-s and unlimited plank tests (time to perform the test for: partial curl-ups (Fitnessgram) = 29 s ± 28, partial curl-ups (Canadian Health Measures Survey (CHMS)) = 36 s ± 21, 60-s plank = 41 s ± 16, 90-s plank = 52 s ± 25 and unlimited plank = 56 s ± 40; zero scores (i.e., floor effect) for: partial curl-ups (Fitnessgram) = 12% of children could not perform the test, partial curl-ups (CHMS) = 5% of children could not perform the test (both tests only differentiated 70% of children, who have lower levels of fitness and are less influenced by skill, technique or previous experience with the test protocols, versus those who have better trunk muscle strength), 60-s plank = only differentiates 55% of children, 90-s plank = only differentiates 86% of children, and for unlimited plank 133 s was required to provide a nonzero/nonmaximal score and to differentiate among 95% of the participants).

One study [44] reported that trunk flexor endurance and isometric back endurance tests were feasible (100% of the participants performed the test, 95.5% of the participants performed the trunk flexor endurance test within the given time (≤ 5 min) and 89% of the participants performed the isometric back endurance test within the given time (≤ 5 min)) in children. Finally, one study [20] reported that isometric back endurance test was feasible (time to prepare and perform the test on a 5 points scale: mean score = 4.3–4.7 (excellent) and 95% of the participants performed the test) in adults.

One high-quality study [33] assessed the feasibility of lower body strength-endurance (see Supplementary Tables S5 and S8). This study found that the 30-s sit-to-stand test was feasible in older adults (100% of the participants performed the test and the time to perform the test was 30 s).

Two high-quality studies [20, 33] analysed the feasibility of upper body strength- endurance tests (see Supplementary Tables S5 and S8). One study [20] reported that the modified push-ups test was feasible (time to prepare and perform the test on a 5 points scale: mean score = 4.3–4.7 (excellent) and 95% of the participants performed the test) in adults. Also, one study showed that the arm curl test [33] was feasible (100% of the participants performed the test) in older adults.

One low-quality study [28] observed that the 45-s squat test was feasible (100% of the participants found the tests to be “low effort” and the test was considered easy to administer) in children and adolescents.

Finally, one very low-quality study [27] found the modified 30-s sit-to-stand test (82% of the participants performed at least 1 repetition of the test) to be feasible in older adults.

#### Flexibility

Two high-quality studies [32, 33] assessed the feasibility of flexibility tests (see Supplementary Tables S5 and S8). One study [32] reported that the sit and reach test was feasible (100% of the participants performed the test) in children. One study [33] showed that the chair sit and reach and the back scratch tests, which assess lower body flexibility and upper body flexibility, were feasible (100% of the participants performed the test) in older adults, respectively.

#### Motor Fitness

##### Speed

Only one high-quality study [36] evaluated the feasibility of the 20-m run test was feasible (100% of the participants performed the test, 100% understood the test instruction and it was considered easy to administer) in children (see Supplementary Tables S5 and S8).

##### Agility

Four high-quality studies [32, 33, 36, 38] evaluated the feasibility of agility tests (see Supplementary Tables S5 and S8). Two studies [32, 38] reported that the 4 × 10-m shuttle run test was feasible (100% of the participants performed the test, time to perform the test was for males = 17.3 ± 2.5 and for females = 18.7 ± 3.1 min [32]; 8 to 10 min to perform the test, maximum 8 participants per group, 2 evaluators assessed the performance of the test, 100% of the participants understood the test instructions and the test was considered easy to administer [38] in children. One study [36] observed that the 10 × 5-m shuttle run test was feasible (100% of the participants performed the test, and understood the test instructions, and the test was considered easy to administer) in children. One high-quality study [33] found that the 2.45-m timed up & go test (also known as the 8-ft up and go test), which assesses agility, balance and speed of walking, was feasible (100% of the participants performed the test) in older adults.

##### Balance

Three high-quality studies [20, 38, 42] evaluated the feasibility of balance tests (see Supplementary Tables S5 and S8). Two studies [38, 42] reported that the single-leg stand test was feasible (8 to 10 min to perform the test, maximum 8 participants per group, 100% of the participants understood the test instructions and the test was considered easy to administer [38]; 100% of the participants performed the test and understood the test instructions, 76% of participants wore appropriate sport clothes, the test was considered easy to administer and the space to perform it was appropriate [42] in children. Another study [20] observed that the single-leg stand test was feasible (time to prepare and perform the test on a 5 points scale: mean score = 4.3–4.7 (excellent), and 95% of the participants performed the test) in adults. Finally, one study [42] showed that the dynamic balance test was feasible (100% of the participants performed the test and understood the test instructions, 76% of participants wore appropriate sport clothes, the test was considered easy to administer and the space to administer the test was appropriate) in children.

##### Multi-skills

One high-quality study [36] showed that jumping a distance of 7-m on 1 foot and jumping a distance of 7-m on 2 feet tests, which assess multiple skills (i.e., agility, balance, coordination, power and speed), were feasible (100% of participants performed the tests, 100% of participants understood the tests instructions and the tests were considered easy to administer) in children.

### Safety

A synthesized version of Supplementary Table S6 is presented as Supplementary Table S9 for easy reading and condensation of the results.

#### Cardiorespiratory Fitness

##### Distance and Time-Based Run/Walk Tests

Four high-quality studies [20, 29, 34, 35] analysed the safety of the distance and time-based run/walk test (see Supplementary Tables S6 and S9). Three studies [20, 34, 35] showed that the 2-km walk test was safe in adults (delayed onset muscle soreness (DOMS) males = 60% and females = 78%, HR bpm males = 153 ± 17.4 and females = 151 ± 18.3, exertion 85%HRmax males = 43% and females = 37%, and ≥ 95% performed the test without adverse events (i.e., sick feeling, pain and/or injuries) [20]; rating of perceived exertion (RPE) males = 3 ± 1.8 and females = 2.9 ± 1.5 (exertion perceived as “moderate”), HR bpm males = 153.1 ± 22.2 and females = 153.8 ± 16 and ≥ 70% performed the test without adverse events (i.e., sick feeling and pain) [34]; RPE = 3.1 ± 2.4 (exertion perceived as “moderate”), HR bpm males = 139.1 ± 19.1 and females = 143.5 ± 17.1 [35]. One study [29] reported that the 6-min walk test was safe (HR bpm = 128 ± 25, Borg scale dyspnea = 2 (1–3) and Borg scale leg fatigue = 2 (1–3) and 100% of participants perform the test without adverse events (i.e., shortness of breath and/or sick feeling)) in adults.

##### 20-m Shuttle Run Test

Two high-quality studies [31, 39] analysed the safety of the 20-m shuttle run test, only in children and adolescents (see Supplementary Tables S6 and S9). One study [39] observed that the 20-m shuttle run test was safe (≥ 99% participants performed the test without adverse events (i.e., sick feeling, pain and/or injuries) and 71.2% experienced some degree of DOMS; of these, in 29.3% it was assumed that the 20-m shuttle run test could be the cause) in children and adolescents. Another study [31] compared the 20-m shuttle run test with music vs. the original version; both tests were considered safe (RPE = 8.3 ± 2 vs. 7.9 ± 2.2 and HR bpm = 177.1 ± 30.6 vs. 177.7 ± 29.2) in adolescents.

Only one low-quality study [32] reported that the 20-m shuttle run test was safe (100% of participants performed the test without adverse events (i.e., sick feeling or injuries)) in children.

##### Step Tests

Three high-quality studies [28, 29, 40] analysed the safety of the step tests (see Supplementary Tables S6 and S9). One study [28] observed that the 3-min step test was safe (RPE = 2.7 ± 1.4 and 100% of participants perform the test without adverse event) in children. Two studies [29, 40], showed that the Chester step (Borg scale dyspnea = 2 (1–3), Borg scale leg fatigue = 2 (1–3), HR bpm = 143 ± 28 and 100% of participants perform the test without adverse events (i.e., shortness of breath and/or sick feeling)); modified incremental step (Borg scale dyspnea = 2 (1–3), Borg scale leg fatigue = 2 (1–4), HR bpm = 142 ± 27 and 100% of participants perform the test without adverse event [29] and Danish step tests (0.1% of participants experienced adverse events (1 participant had strained calf after 30 s)) were safe in adults [40].

Only one low-quality study [30] reported that the 6-min step test was safe (Borg scale dyspnea = 2.5 ± 1.5, Borg scale leg fatigue = 3.4 ± 2.5 and HR bpm = 118.2 ± 18.7) in adults.

#### Muscular Strength

##### Maximal Isometric Strength

Three high-quality studies [20, 39, 41] analysed the safety of hand maximal isometric strength, using the handgrip strength test (see Supplementary Tables S6 and S9). Two studies [39, 41] concluded that the test was safe (100% of participants performed the test without adverse events (i.e., pain or discomfort) [41]; ≥ 99% participants performed the test without adverse events (i.e., sick feeling and pain in hand or forearm) and instrument allergy, and 0% experienced DOMS [39] in children and adolescents. One study [20] reported that the handgrip strength test was safe (DOMS males and females = 0%, HR bpm males = 95 ± 15.2 and females = 92 ± 15.3, exertion 85%HRmax = 0% (participants exceeded the maximum percentage) and ≥ 95% of participants performed the test without adverse events (i.e., pain and/or injuries)) in adults.

Only one low-quality study [32] found that the handgrip strength test was safe (100% of participants perform the test without adverse events (i.e., pain or injuries)) in children.

##### Explosive Strength

Three high-quality studies [20, 39, 42] assessed the safety of lower body explosive strength tests (see Supplementary Tables S6 and S9). Two studies [39, 42] showed that the standing long jump test was safe (100% of the participants performed the test without adverse events (i.e., shortness of breath, sick feeling, falls, pain and/or injuries) [42]; ≥ 99% participants performed the test without adverse events (i.e., sick feeling and injury) and 71.2% experienced some degree of DOMS between differences that could be the cause (i.e., 20-m shuttle run or standing long jump tests) [39] in children and adolescents. One study [20] reported that the vertical jump test was safe (HR bpm males = 110 ± 16.3 and females = 112 ± 17.4, exertion 85%HRmax = 0% (participants exceeded the maximum percentage) and ≥ 95% performed the test without adverse events (i.e., sick feeling, pain and/or injuries)) in adults.

Only one low-quality study [32] observed that the standing long jump test was safe (100% of the participants perform the test without adverse events (i.e., pain or injuries)) in children.

##### Strength-Endurance

Two high-quality studies [20, 44] analysed the safety of trunk strength-endurance tests (see Supplementary Tables S6 and S9). The trunk flexor endurance and isometric back endurance tests were safe (100% of participants performed the test without adverse events (i.e., pain and/or injuries)) [44]; HR bpm males = 112 ± 18.3 and females = 121 ± 19.8, exertion 85%HRmax = 1.28% (participants exceeded the maximum) and ≥ 90% performed the test without adverse events (i.e., sick feeling, pain and/or injuries) [20] in adults.

One high-quality study [28] analysed the safety of lower body strength-endurance tests (see Supplementary Tables S6 and S9). The 45-s squat test was safe (RPE = 3.4 ± 1.7 and 100% of participants perform the test without adverse event (i.e., sick feeling, joint pain or any tripping or falls)) in children.

One high-quality study [20] analysed the safety of upper body strength-endurance tests (see Supplementary Tables S6 and S9). The modified push-ups test was safe (HR bpm males = 140 ± 18 and females = 144 ± 17.5, exertion 85%HRmax = 9.2% (participants exceeded the maximum) and ≥ 90% performed the test without adverse events (i.e., sick feeling, pain and/or injuries)) in adults.

#### Motor Fitness

##### Balance

Two high-quality studies [20, 42] evaluated the safety of the balance tests (see Supplementary Tables S6 and S9). One study [42] observed that the dynamic balance and the single-leg stand tests were safe (100% of participants performed the test without adverse events (i.e., shortness of breath, sick feeling, falls, pain and/or injuries)) in children. Another study [20] found that the single-leg stand test was safe (HR bpm males = 100 ± 16.1 and females = 94 ± 16, exertion 85%HRmax = 0% (participants exceeded the maximum) and ≥ 90% performed the test without adverse events (i.e., sick feeling, pain and/or injuries)) in adults.

### Overview of Evidence of Criterion-Related Validity, Reliability, Feasibility and Safety of Field-Based Physical Fitness Tests in Adults

A comprehensive view of levels of evidence of criterion-related validity, reliability, feasibility and safety of field-based physical fitness tests in adults are shown in Tables 3 and 4. All the field-based physical fitness tests were found to have an overall or combined limited evidence. Of the field-based physical fitness tests that showed strong or moderate evidence for criterion-related validity and reliability had overall or combined limited evidence due to a lack of evidence of their feasibility and/or safety.

## Discussion

The present systematic review comprehensively studied the feasibility and the safety of the existing field-based physical fitness tests used in people of all ages and provide an evidence-based proposal for the most feasible and safe field-based physical fitness tests. This study complements previous studies of the validity [22, 23] and the reliability [21, 24] of field-based physical fitness tests have been widely studied in children, adolescents and adults.

This review shows that few studies have addressed the feasibility and the safety of field-based physical fitness tests in children and adolescents, adults or/and older adults. Only the 2-km walk test in adults and 20-m shuttle run, handgrip strength and standing long jump tests in children and adolescents showed strong evidence on feasibility (see Table 1). However, there was limited evidence in older adults. Thus, there was no homogeneity in the study of the feasibility of testing by age group. Regarding safety, only the 2-km walk test was shown to have strong evidence in adults. The evidence presented was only in youth and adults. The evidence was only analysed in 11 studies (see Table 2). Therefore, we found that most field-based physical fitness tests, which are often widely used in one or more age groups, were not shown to have strong or moderate supporting evidence. Based on these findings, more studies are needed to determine the feasibility and the safety of field-based physical fitness tests in people of all ages.

Moreover, we have analysed the combined evidence on feasibility and safety, determined by in this study, and criterion-related validity and reliability, as previously reviewed [23, 24], of field-based physical fitness tests in the adult population. Overall, all tests were found to have limited evidence, mainly due to the lack of evidence on feasibility and safety.

### Levels of evidence

#### Feasibility

##### Cardiorespiratory Fitness

Strong evidence indicated that the 2-km walk and 20-m shuttle run tests were feasible in adults and children and adolescents, respectively. Limited evidence showed that the 6-min run test was feasible in children and adolescents, the Danish step test in adults, and the 6-min walk and 2-min step in older population, were feasible.

##### Muscular Strength

Strong evidence indicated that (a) the handgrip strength test was feasible in children and adolescents; and (b) the standing long jump test was feasible in children and adolescents. Moderate evidence indicated that the isometric back endurance test was feasible in adults. Limited evidence indicated that (a) the handgrip strength test was feasible in adults; (b) the vertical jump test was feasible in adults; (c) the medicine ball push test was feasible in children and adolescents; (d) the 60-s, 90-s and unlimited plank tests in children and adolescents; the trunk flexor endurance test in adults; and the partial curl-ups test in children and adolescents, were feasible; (e) the 30-s sit-to-stand test was feasible in older adults; (f) the arm curl test in older adults; and the modified push-ups test in adults, were feasible.

##### Flexibility

Limited evidence indicated that the sit and reach test in children, the chair sit and reach in older adults and the back scratch test in older adults, were feasible.

##### Motor Fitness

Moderate evidence indicated that (a) the 4 × 10-m shuttle run test was feasible in children and adolescents; and (b) the single-leg stand test was feasible in children and adolescents. Limited evidence indicated that (a) the 20-m run test was feasible in children and adolescents; (b) the 10 × 5-m shuttle run test in children and adolescents, and the 2.45-m timed up & go test in older adults were feasible; (c) the dynamic balance test in children and adolescents, and the single-leg stand test in adults were feasible; (d) the jumping a distance of 7-m on 1 foot and jumping a distance of 7-m on 2 feet tests in children and adolescents were feasible.

#### Safety

##### Cardiorespiratory Fitness

Strong evidence indicated that only the 2-km walk test was safe in adults. Moderate evidence indicated that the 20-m shuttle run test was safe in children and adolescents. Limited evidence indicated that the 6-min walk test in children and adolescents, the Danish step test in adults, the 3-min step test in children and adolescents, the Chester step and modified incremental step tests in adults.

##### Muscular Strength

Moderate evidence indicated that (a) the handgrip strength test was safe in children and adolescents; (b) the standing long jump test was safe in children and adolescents; and (c) the isometric back endurance test was safe in adults. Limited evidence indicated that (a) the handgrip strength test was safe in adults; (b) the vertical jump test was safe in adults; (c) the trunk flexor endurance test was safe in adults; (d) the modified push-ups test was safe in adults; and (e) the 45-s squat test was safe in children and adolescents.

##### Flexibility

No studies examined the safety of the flexibility field-based physical fitness tests; therefore, null evidence was found.

##### Motor Fitness

Limited evidence indicated that (a) the dynamic balance test was safe in children and adolescents; and (b) the single-leg stand test was safe in children and adolescents and adults.

It is important to highlight that analysing the levels of evidence for each test by age groups, we observed no evidence was found in safety studies for the older adult population.

The evidence gathered allows us to observe that most studies have analysed the feasibility and safety of field-based physical fitness tests mainly in children and adolescents, for whom there is already a health-related fitness test battery, with evidence-based on their criterion-related validity, reliability, feasibility and safety [45]. In adults, less evidence is available and the results are inconsistent in terms of feasibility and safety. Only the 2-km walk test has shown strong evidence on feasibility and safety, and previous systematic review have shown strong evidence of its criterion-related validity [23], but its reliability is limited due to the low number of studies in this population [24] (see Tables 3 and 4). Finally, there is limited evidence in older adults, which highlights the need for more studies in this population.

Analyzing the combined evidence of criterion-related validity, reliability, feasibility and safety in adults, all field-based physical fitness tests have limited evidence, mostly due to a lack of evidence on feasibility and safety. Thus, our review highlights the need for more studies that assess the feasibility and safety of field-based physical fitness tests to complement the existing validity and reliability studies. Ultimately, the aim is to propose a health-related field-based physical fitness tests in adults, based on its validity (i.e., predictive and criterion-related), reliability, feasibility, and safety, and in addition, responsiveness.

### Criteria to Assess Feasibility and Safety

Homogeneous criteria were not observed when assessing the field-based physical fitness test feasibility and safety, which could influence the final results.

There was no consensus on which items were appropriate to assess feasibility, and whether they should have been reported by the participants and/or evaluators (see Supplementary Table S8). Three studies included items reported by participants [28, 31, 35], all studies, except Lamoneda et al. [31], included items reported by evaluators, and two studies used a mixed evaluation [28, 35], with more items based on the perception of the evaluators and/or participants, than objective items.

Among the items reported to assess feasibility by participants, the “self-perception effort” [28, 31, 35] was the most used. “Self-perception fatigue”, “self-perception preference test” and “self-perception enjoyment” were the other items reported by the participants, which were used only once in a single study [31]. It is important to highlight that the “self-perception effort” and “self-perception fatigue” items are more related to the safety assessment. In fact, they have been used in most of the safety studies included in this systematic review, which corroborates that there is no unanimity of criteria. Likewise, Hébert et al. [41], included the item “adverse event” to assess feasibility, when it is an item used by most authors to assess safety.

The items most frequently recorded by evaluators were “time to perform the test” used in 16 studies [20, 27–30, 32–35, 37–40, 42–44], “participants performing the test” in 13 studies [20, 27, 30, 32–36, 40–44], “easy to administer” in seven studies [28, 36, 38, 39, 41, 42, 44], “number of evaluators” in five studies [20, 35, 37–39], and “understand test instructions” in four studies [36, 39, 41, 42].

It can be observed that these items reported by evaluators, were mostly focused on the time necessary for the preparation, development and implementation of the test, which varied depending on the appropriate facilities and equipment, test procedures, number of tests assessed, number of testers and number of participants that can be tested at the same time. Only two studies [37, 39] provided information about the time for preparation, number of evaluators, number of participants assessed by test and time to perform the test/s. This information would be interesting when choosing a test according to its feasibility, and designing future field-based physical fitness test batteries. Interestingly, only two studies have explicitly assessed whether sport clothes and facilities were appropriate or not [39, 42]. Studies that did not include these items might reflect an assumption that in order to administer a field-based physical fitness test, sports clothing and facilities must be appropriate.

Regarding the total number of feasibility items included in each study, only one study included nine items assessed by evaluators [39], one study used six items [42], and five items [35], three studies used four items [31, 37, 38] and 13 studies (68.4%) only used between one and three items [20, 27–30, 32–34, 36, 40, 41, 43, 44].

Similarly, safety was assessed with items reported by evaluator and participants (see Supplementary Table S9). Most of the studies used items reported by participants and evaluators [20, 28–31, 34, 35, 39], except five studies that used only items reported by evaluators [32, 40–42, 44]. Note that these five studies only used the “adverse events” item to assess safety.

We only found two objective items based on “HR” and “exertion 85% HRmax” which were included in seven studies [20, 28–31, 34, 35]. The “HR” item together with “adverse events” [20, 28, 29, 32, 34, 39–42, 44] and “self-perception general exertion” items [28, 29, 31, 34, 35] were the most used to assess safety. Most of the items were related to the perception of effort/fatigue or feeling sick (i.e., self-perceived dyspnea and fatigue, general exertion, DOMS and sick feeling). Only one study [39] included a questionnaire related to “instrument allergy”.

HR assessed at the end of the test was the most commonly used safety item in cardiorespiratory fitness tests (i.e., the 2-km walk, 6-min walk, 20-m shuttle run, 45-s squat, 3-min step, Chester step, modified incremental step and 6-min step tests). Instead of using the absolute HR as a safety item, we suggest using the relative value as a percentage of HRmax or similar to assess safety. Only one study [20] employed this last item, expressing the exertion as the percentage of age-predicted HRmax, establishing 85% as the cut-off point. On the other hand, “adverse events” was the most used item to evaluate the safety of cardiorespiratory fitness (i.e., 20-m shuttle run, 45-s squat, 3-min step, Chester step, modified incremental step, Danish step, 2-km walk tests), musculoskeletal fitness tests (i.e., the handgrip strength, vertical jump, standing long jump, isometric back endurance, modified push-ups and trunk flexor endurance), and motor fitness tests (i.e., the dynamic balance and single-leg stand).

Of the 11 items proposed in the studies to assess safety, two studies used four items [29, 39], and 11 studies (85% of the studies) only utilized between one and three items [28, 30–32, 34, 35, 39–42, 44].

It has been observed that no established analyses exist to assess the feasibility and safety of the field-based tests (see Supplementary Tables S8 and S9). All the studies used the frequency in number, mean or percentages to decide whether the items evaluated did or did not meet the feasibility and safety criteria. However, a value or cut-off point was not established to determine whether or not an item meets the feasibility and safety criteria. Only España-Romero et al. [39] considered that a field-based physical fitness test met the feasibility and safety criteria when the items were positively answered in at least 95% and 99%, respectively. Thus, there was no consensus on whether or not an item met the criteria for feasibility or safety. All these differences when assessing, analysing and concluding on the findings, could lead to biases when presenting the results and objectively establishing that a study is feasible and/or safe to measure physical fitness in people of all ages, especially if there are no valid tools designed to assess them.

Finally, based on our findings, there are items that should be considered as a high priority to assess the feasibility and safety of field-based physical fitness test in children, adolescents, adults or/and older adults. We consider that to assess feasibility we should use at least the following items: (a) “time to perform the test”; (b) “time to prepare the test”; (c) “percentage of rejection”; (d) “percentage of participants who performed the test”; (e) “number of participants measured by tests”; (f) “number of evaluators”; (g) “easy to administer”; and (h) “percentage of participants who understood test instructions”. Appropriate facilities, equipment and sportswear must be assumed as a condition, whenever we assess field-based physical fitness test. Regarding the assessment of safety, we highlight (a) “exertion %HRmax”; (b) “percentage of adverse events” (i.e., falls, sick feeling, pain and/or injur(y); (c) “general exertion and/or fatigue; (d) “DOMS”; and (e) “instrument allergy”, in cases where we used instruments that were in direct contact with the participant’s skin.

**Fig. 2:**
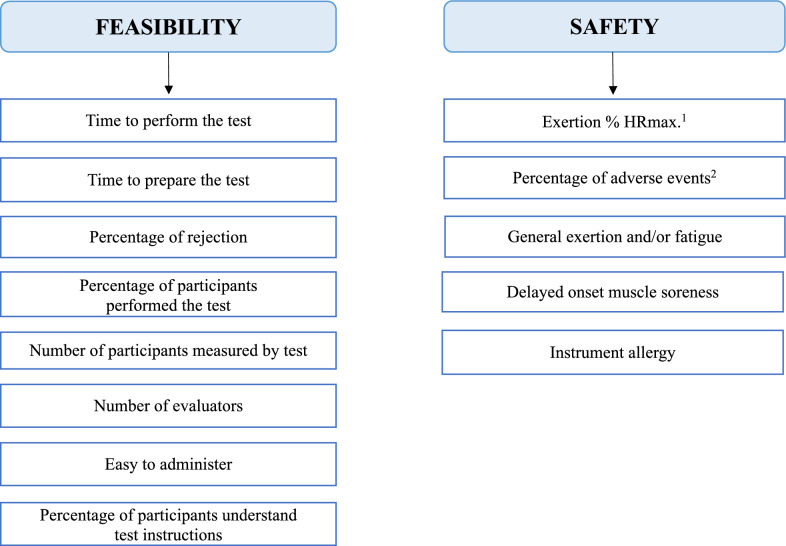
Proposed items for assessing the feasibility and the safety of field-based physical fitness tests in people of all ages. ^1^The exertion % HR max. will depends on the maximum and submaximal characteristics of the test.^2^Adverse events refer to falls, sick feeling, pain and/or injury

## Conclusions

To our knowledge, this is the first systematic review to examine the feasibility and safety of field-based physical fitness test at all ages. There are few studies that have analysed the feasibility and safety of field-based physical fitness tests, and overall, there is little evidence. Most of the tests analysed showed limited evidence, due to the lack of studies, especially in older adults. Based on quality assessment and established levels of evidence for each study, our findings indicate: strong evidence indicated that (a) the 2-km walk and 20-m shuttle run tests were feasible in adults and children and adolescents, respectively; (b) the handgrip strength test was feasible in children and adolescents; and (c) the standing long jump test was feasible in children and adolescents. Regarding safety, only the 2-km walk test showed strong evidence in adults.

There is no consensus on the assessment of feasibility and safety of field-based physical fitness tests. It would be desirable that experts in this field generate methodological criteria that could correctly reflect the feasibility or/and safety of field-based physical fitness tests in people of all ages. With this in mind, we have made a proposal of the items that we consider should be used to assess the feasibility and safety of field-based physical fitness tests (Fig. 2).

The combined evidence on criterion-related validity, reliability, feasibility and safety of field-based tests was found to be limited in adults due to the lack of evidence on feasibility and safety, highlighting the need for additional studies to complement the existing evidence in this population, as well as in older adults.

## Supplementary Information

Supplementary Material 1.

Supplementary Material 2.

Supplementary Material 3.

Supplementary Material 4.

Supplementary Material 5.

Supplementary Material 6.

Supplementary Material 7.

Supplementary Material 8.

Supplementary Material 9.

Supplementary Material 10.

## Data Availability

The authors declare that all relevant data are included in the article and/or its supplementary information files.

## Abbreviations

BMI: Body mass index
CHMS: Canadian health measure survey
CVD: Cardiovascular disease
DOMS: Delayed-onset muscle soreness
HR: Heart rate
MeSH: Medical subject heading
PRISMA: Preferred reporting items for systematic reviews and meta-analysis
PROSPERO: International prospective register of systematic reviews
QoL: Quality of life
RPE: Rating of perceived exertion
