# Supplementary material for: Feasibility and Safety of Field-Based Physical Fitness Tests: A Systematic Review
Source: Sports Med Open. 2025 Jan 24;11:8. doi: 10.1186/s40798-024-00799-1 (PMC11759754; doi:10.1186/s40798-024-00799-1)
Supplement: Supplementary file 1 — Supplementary Material 1. [file 40798_2024_799_MOESM1_ESM.docx]

**Suplementary Material 1. Search strategy terms**

**MEDLINE (via PubMed):** Separated searches

(feasibility [All Fields] OR viability [All Fields] OR safety [All Fields]) AND (“Physical fitness"[Mesh])

(feasibility [All Fields] OR viability [All Fields] OR safety [All Fields]) AND ("Muscle Strength"[ Mesh] OR "Muscle strength dynamometer"[Mesh])

(feasibility [All Fields] OR viability [All Fields] OR safety [All Fields]) AND (“Range of motion, articular” [Mesh Major Topic])

(feasibility [All Fields] OR viability [All Fields] OR safety [All Fields]) AND ("Postural Balance"[MeSH])

(feasibility [All Fields] OR viability [All Fields] OR safety [All Fields]) AND (“Physical Endurance"[ MeSH])

(feasibility [All Fields] OR viability [All Fields] OR safety [All Fields]) AND (“Cardiorespiratory fitness” [All Fields] OR “Cardiovascular fitness” [All Fields] OR “Aerobic fitness” [All Fields] OR “Aerobic capacity” [All Fields] OR “Maximal oxygen consumption” [All Fields] OR “VO_2_max” [All Fields])

(feasibility [All Fields] OR viability [All Fields] OR safety [All Fields]) AND ("Motor fitness"[ All Fields])

(feasibility [All Fields] OR viability [All Fields] OR safety [All Fields]) AND ("Running Speed"[All Fields])

(feasibility [All Fields] OR viability [All Fields] OR safety [All Fields]) AND (Agility [All Fields])

**PubMed:** All searches combined

(((((((((feasibility [All Fields] OR viability [All Fields] OR safety [All Fields]) AND ("Physical fitness"[Mesh])) OR ((feasibility [All Fields] OR viability [All Fields] OR safety [All Fields]) AND ("Muscle Strength"[ Mesh] OR "Muscle strength dynamometer"[Mesh]))) OR ((feasibility [All Fields] OR viability [All Fields] OR safety [All Fields]) AND ("Range of motion, articular"[Mesh Major Topic]))) OR ((feasibility [All Fields] OR viability [All Fields] OR safety [All Fields]) AND ("Postural Balance"[MeSH]))) OR ((feasibility [All Fields] OR viability [All Fields] OR safety [All Fields]) AND ("Physical Endurance"[ MeSH]))) OR ((feasibility [All Fields] OR viability [All Fields] OR safety [All Fields]) AND ("Cardiorespiratory fitness"[All Fields] OR "Cardiovascular fitness"[All Fields] OR "Aerobic fitness"[All Fields] OR "Aerobic capacity"[All Fields] OR "Maximal oxygen consumption"[All Fields] OR "VO2max"[All Fields]))) OR ((feasibility [All Fields] OR viability [All Fields] OR safety [All Fields]) AND ("Motor fitness"[ All Fields]))) OR ((feasibility [All Fields] OR viability [All Fields] OR safety [All Fields]) AND ("Running Speed"[All Fields]))) OR ((feasibility [All Fields] OR viability [All Fields] OR safety [All Fields]) AND (Agility [All Fields]))

**Web of Sciences:** Searches

(feasibility OR viability OR safety) AND ("Physical fitness" OR "Physical Conditioning")

(feasibility OR viability OR safety) AND ("Muscle strength" OR "Muscular strength" OR dynamometer*)

(feasibility OR viability OR safety) AND (“Joint Range of Motion” OR “Joint flexibility” OR “Range of motion”) AND (Test)

(feasibility OR viability OR safety) AND (“Musculoskeletal Equilibrium” OR “Postural Balance” OR “Postural Equilibrium”)

(feasibility OR viability OR safety) AND (“Cardiorespiratory fitness” OR “Cardiovascular fitness” OR “Aerobic fitness” OR “Aerobic fitness” OR “Aerobic capacity” OR “Maximal oxygen consumption” OR “VO2max” OR "Physical Endurance")

(feasibility OR viability OR safety) AND (“Running Speed” OR “Agility” OR "Motor fitness")

**Web of Sciences:** Separated by physical condition

TS=(feasibility OR viability OR safety) AND TS=("Physical fitness" OR "Physical Conditioning")

TS=(feasibility OR viability OR safety) AND TS=(“Joint Range of Motion” OR “Joint flexibility” OR “Range of motion”) AND TS=(Test)

TS=(feasibility OR viability OR safety) AND TS=("Muscle strength" OR "Muscular strength" OR dynamometer*)

TS=(feasibility OR viability OR safety) AND TS=("Musculoskeletal Equilibrium” OR “Postural Balance” OR “Postural Equilibrium”)

TS=(feasibility OR viability OR safety) AND TS=(“Cardiorespiratory fitness” OR “Cardiovascular fitness” OR “Aerobic fitness” OR “Aerobic fitness” OR “Aerobic capacity” OR “Maximal oxygen consumption” OR “VO2max” OR "Physical Endurance")

TS=(feasibility OR viability OR safety) AND TS=(“Running Speed” OR “Agility” OR "Motor fitness")

**Web of Sciences:** All searches combined

TS=(feasibility OR viability OR safety) AND TS=("Physical fitness" OR "Physical Conditioning" OR "Muscle strength" OR "Muscular strength" OR dynamometer* OR "Musculoskeletal Equilibrium” OR “Postural Balance” OR “Postural Equilibrium” OR “Cardiorespiratory fitness” OR “Cardiovascular fitness” OR “Aerobic fitness” OR “Aerobic fitness” OR “Aerobic capacity” OR “Maximal oxygen consumption” OR “VO2max” OR "Physical Endurance" OR “Running Speed” OR “Agility” OR "Motor fitness" OR “Joint Range of Motion” OR “Joint flexibility” OR “Range of motion”) AND TS=(Test)
