# Supplementary material for: Feasibility and Safety of Field-Based Physical Fitness Tests: A Systematic Review
Source: Sports Med Open. 2025 Jan 24;11:8. doi: 10.1186/s40798-024-00799-1 (PMC11759754; doi:10.1186/s40798-024-00799-1)
Supplement: Supplementary file 2 — Supplementary Material 2. [file 40798_2024_799_MOESM2_ESM.docx]

**Supplementary Table S1.** Quality assessment list for field-based fitness test feasibility studies.

| Grading system parameter | Grade | Criterion |
| --- | --- | --- |
| Number of study subjects | 0 | n ≤ 10 |
|  | 1 | n = 11-50 |
|  | 2 | n ≥ 51 |
| Description of the study population  with respect to age, sex, health status,  fitness levels, ethnicity, physical activity patterns, body composition, etc. | 0 | Less items than required for grade 1 |
|  | 1 | At least age, sex, health status, and fitness levels |
|  | 2 | More items than required for grade 1 |
| Number of items reported: time spent preparing and developing the test, percentage of participants completing the test, and self-reporter feasibility questions to participants/evaluators | 0 | Less items than required for grade 1 |
|  | 1 | At least time spent preparing and developing the test, AND percentage of participants completing the test OR self-reporter questions to participants/evaluators |
|  | 2 | Time spent preparing and developing the test, percentage of participants completing the test and self-reporter questions to participants/evaluators |

Rating for total score: high quality = 5–6; low quality = 3–4; very low quality = 0–2.
