# Supplementary material for: Feasibility and Safety of Field-Based Physical Fitness Tests: A Systematic Review
Source: Sports Med Open. 2025 Jan 24;11:8. doi: 10.1186/s40798-024-00799-1 (PMC11759754; doi:10.1186/s40798-024-00799-1)
Supplement: Supplementary file 3 — Supplementary Material 3. [file 40798_2024_799_MOESM3_ESM.docx]

**Supplementary Table S2.** Quality assessment list for field-based fitness test safety studies.

| Grading system parameter | Grade | Criterion |
| --- | --- | --- |
| Number of study subjects | 0 | n ≤ 10 |
|  | 1 | n = 11-50 |
|  | 2 | n ≥ 51 |
| Description of the study population  with respect to age, sex, health status,  fitness levels, pubertal status, ethnicity, physical activity patterns, body composition, etc. | 0 | Less items than required for grade 1 |
|  | 1 | At least age, sex, health status, and fitness levels |
|  | 2 | More items than required for grade 1 |
| Number of questions/ items reported: adverse events (i.e., instrument allergy, sick feeling, pain, musculoskeletal injuries, falls...), delayed-onset muscle soreness, heart rate, Borg rating of shortness of breath, etc. | 0 | Less that required for grade 1 or 2 |
|  | 1 | At least adverse events |
|  | 2 | At least adverse events, AND delayed-onset muscle soreness OR heart rate OR Borg rating of shortness of breath |

Rating for total score. high quality = 5-6; low quality = 3-4; very low quality = 0– 2.
