# Supplementary material for: Feasibility and Safety of Field-Based Physical Fitness Tests: A Systematic Review
Source: Sports Med Open. 2025 Jan 24;11:8. doi: 10.1186/s40798-024-00799-1 (PMC11759754; doi:10.1186/s40798-024-00799-1)
Supplement: Supplementary file 4 — Supplementary Material 4. [file 40798_2024_799_MOESM4_ESM.docx]

**Supplementary Table S3.** Quality assessment of field-based fitness test feasibility studies.

| Study | Fitness Component | Field-based fitness test | Number of study subjects | Description of the study population | Statistical analysis | Total score |
| --- | --- | --- | --- | --- | --- | --- |
| Borel et al., 2010^[30]^ | Cardiorespiratory fitness | 6-min step | 1 | 2 | 0 | 3 |
| Bruggeman et al., 2020^[28]^ | Cardiorespiratory fitness | 3-min step | 1 | 2 | 1 | 4 |
| Anderson & Dal Corso, 2016^[29]^ | Cardiorespiratory fitness | Chester step | 1 | 2 | 1 | 4 |
| Anderson & Dal Corso, 2016^[29]^ | Cardiorespiratory fitness | Modified incremental step | 1 | 2 | 1 | 4 |
| Anderson & Dal Corso, 2016^[29]^ | Cardiorespiratory fitness | 6-min walk | 1 | 2 | 1 | 4 |
| Lamoneda et al., 2020^[31]^ | Cardiorespiratory fitness | 20-m shuttle run music | 2 | 1 | 1 | 4 |
| Lamoneda et al., 2020^[31]^ | Cardiorespiratory fitness | 20-m shuttle run | 2 | 1 | 1 | 4 |
| Oja et al., 1991^[34]^ | Cardiorespiratory fitness | 2-km walk | 2 | 2 | 1 | 5 |
| Amado-Pacheco et al., 2019^[32]^ | Cardiorespiratory fitness | 20-m shuttle run | 2 | 2 | 1 | 5 |
| Cadenas-Sanchez et al., 2014^[37]^ | Cardiorespiratory fitness | 20-m shuttle run | 2 | 2 | 1 | 5 |
| Cadenas-Sánchez et al., 2016^[38]^ | Cardiorespiratory fitness | 20-m shuttle run | 2 | 2 | 1 | 5 |
| Fjortoft et al., 2011^[36]^ | Cardiorespiratory fitness | 6-min run | 2 | 2 | 1 | 5 |
| Aadahl et al., 2012^[40]^ | Cardiorespiratory fitness | Danish step | 2 | 2 | 2 | 6 |
| Langhammer & Stanghelle, 2018^[33]^ | Cardiorespiratory fitness | 2-min step | 2 | 2 | 2 | 6 |
| Langhammer & Stanghelle, 2018^[33]^ | Cardiorespiratory fitness | 6-min walk | 2 | 2 | 2 | 6 |
| Laukkanen et al., 1992^[35]^ | Cardiorespiratory fitness | 2-km walk | 2 | 2 | 2 | 6 |
| Suni et al., 1998^[20]^ | Cardiorespiratory fitness | 2-km walk | 2 | 2 | 2 | 6 |
| España-Romero et al., 2010^[39]^ | Cardiorespiratory fitness | 20-m shuttle run | 2 | 2 | 2 | 6 |
| McAllister & Palombaro, 2019^[27]^ | Musculoskeletal fitness | Modified 30-s sit-to-stand | 0 | 1 | 1 | 2 |
| Bruggeman et al., 2020^[28]^ | Musculoskeletal fitness | 45-s squat | 1 | 2 | 1 | 4 |
| Hébert et al., 2011^[41]^ | Musculoskeletal fitness | Handgrip | 2 | 2 | 1 | 5 |
| Amado-Pacheco et al., 2019^[32]^ | Musculoskeletal fitness | Handgrip | 2 | 2 | 1 | 5 |
| Cadenas-Sánchez et al., 2016^[38]^ | Musculoskeletal fitness | Handgrip | 2 | 1 | 2 | 5 |
| Fjortoft et al., 2011^[36]^ | Musculoskeletal fitness | Standing long jump | 2 | 2 | 1 | 5 |
| Amado-Pacheco et al., 2019^[32]^ | Musculoskeletal fitness | Standing long jump | 2 | 2 | 1 | 5 |
| Cadenas-Sánchez et al., 2016^[38]^ | Musculoskeletal fitness | Standing long jump | 2 | 1 | 2 | 5 |
| Smits-Engelsman et al., 2020^[42]^ | Musculoskeletal fitness | Standing long jump | 2 | 1 | 2 | 5 |
| Fjortoft et al., 2011^[36]^ | Musculoskeletal fitness | Medicine ball push | 2 | 2 | 1 | 5 |
| Boyer et al., 2013^[43]^ | Musculoskeletal fitness | Partial curl-ups, 60-s, 90-s and unlimited plank | 2 | 2 | 1 | 5 |
| Ito et al., 1996^[44]^ | Musculoskeletal fitness | Trunk flexor endurance | 2 | 2 | 1 | 5 |
| Ito et al., 1996^[44]^ | Musculoskeletal fitness | Isometric back endurance | 2 | 2 | 1 | 5 |
| Amado-Pacheco et al., 2019^[32]^ | Musculoskeletal fitness | Sit and reach | 2 | 2 | 1 | 5 |
| España-Romero et al., 2010^[39]^ | Musculoskeletal fitness | Handgrip | 2 | 2 | 2 | 6 |
| Suni et al., 1998^[20]^ | Musculoskeletal fitness | Handgrip | 2 | 2 | 2 | 6 |
| España-Romero et al., 2010^[39]^ | Musculoskeletal fitness | Standing long jump | 2 | 2 | 2 | 6 |
| Suni et al., 1998^[20]^ | Musculoskeletal fitness | Vertical jump | 2 | 2 | 2 | 6 |
| Langhammer & Stanghelle, 2018^[33]^ | Musculoskeletal fitness | 30-s sit-to-stand | 2 | 2 | 2 | 6 |
| Langhammer & Stanghelle, 2018^[33]^ | Musculoskeletal fitness | Arm curl | 2 | 2 | 2 | 6 |
| Suni et al., 1998^[20]^ | Musculoskeletal fitness | Isometric back endurance | 2 | 2 | 2 | 6 |
| Suni et al., 1998^[20]^ | Musculoskeletal fitness | Modified push-ups | 2 | 2 | 2 | 6 |
| Langhammer & Stanghelle, 2018^[33]^ | Musculoskeletal fitness | Chair sit and reach | 2 | 2 | 2 | 6 |
| Langhammer & Stanghelle, 2018^[33]^ | Musculoskeletal fitness | Back scratch | 2 | 2 | 2 | 6 |
| Fjortoft et al., 2011^[36]^ | Motor fitness | 20-m run | 2 | 2 | 1 | 5 |
| Amado-Pacheco et al., 2019^[32]^ | Motor fitness | 4 × 10-m shuttle run | 2 | 2 | 1 | 5 |
| Cadenas-Sánchez et al., 2016^[38]^ | Motor fitness | 4 × 10-m shuttle run | 2 | 1 | 2 | 5 |
| Fjortoft et al., 2011^[36]^ | Motor fitness | 10 × 5-m shuttle run | 2 | 2 | 1 | 5 |
| Cadenas-Sánchez et al., 2016^[38]^ | Motor fitness | Single-leg stand | 2 | 1 | 2 | 5 |
| Smits-Engelsman et al., 2020^[42]^ | Motor fitness | Single-leg stand | 2 | 1 | 2 | 5 |
| Smits-Engelsman et al., 2020^[42]^ | Motor fitness | Dynamic balance | 2 | 1 | 2 | 5 |
| Fjortoft et al., 2011^[36]^ | Motor fitness | Jumping a distance of 7 m on 1 foot | 2 | 2 | 1 | 5 |
| Fjortoft et al., 2011^[36]^ | Motor fitness | Jumping a distance of 7 m on 2 feet | 2 | 2 | 1 | 5 |
| Langhammer & Stanghelle, 2018^[33]^ | Motor fitness | 2.45-m time up & go | 2 | 2 | 2 | 6 |
| Suni et al., 1998^[20]^ | Motor fitness | Single-leg stand | 2 | 2 | 2 | 6 |

Total score indicates high quality = 5-6; low quality = 3-4; very low quality = 0-2.

**REFERENCES**

20. Suni JH, Miilunpalo, S. I., Asikainen, T. M., Laukkanen, R. T., Oja, P., Pasanen, M. E., & Vuori, I. M. Safety and feasibility of a health-related fitness test battery for adults. Phys Ther. 1998;78(2):134-48.

27. McAllister LS, & Palombaro, K. M. Modified 30-second sit-to-stand test: reliability and validity in older adults unable to complete traditional sit-to-stand testing. J Geriatr Phys Ther. 2020;43(3):153-8.

28. Bruggeman BS, Vincent, H. K., Chi, X., Filipp, S. L., Mercado, R., Modave, F., & Bernier, A. Simple tests of cardiorespiratory fitness in a pediatric population. Plos one. 2020;15(9).

29. José A, & Dal Corso, S. Step tests are safe for assessing functional capacity in patients hospitalized with acute lung diseases. J Cardiopulm Rehabil Prev. 2016;36(1):56-61.

30. Borel B, Fabre, C., Saison, S., Bart, F., & Grosbois, J. M. An original field evaluation test for chronic obstructive pulmonary disease population: the six-minute stepper test. Clin Rehabil. 2010;24(1):82-93.

31. Lamoneda J, Huertas-Delgado, F. J., & Cadenas-Sanchez, C. Feasibility and concurrent validity of a cardiorespiratory fitness test based on the adaptation of the original 20 m shuttle run: The 20 m shuttle run with music. J Sports Sci. 2021;39(1):57-63.

32. Amado-Pacheco JC, Prieto-Benavides DH, Correa-Bautista JE, García-Hermoso A, Agostinis-Sobrinho C, María Alonso-Martínez A., et al. Feasibility and reliability of physical fitness tests among colombian preschool children. Int J Environ Res Public Health. 2019;16(17):3069.

33. Langhammer B, & Stanghelle, J. K. Senior fitness test; a useful tool to measure physical fitness in persons with acquired brain injury. Brain Inj. 2019;33(2):183-8.

34. Oja P, Laukkanen, R., Pasanen, M., Tyry, T., & Vuori, I. A 2-km walking test for assessing the cardiorespiratory fitness of healthy adults. Int J Sports Med. 1991;12(4):356-62.

35. Laukkanen RM, Oja, P., Ojala, K. H., Pasanen, M. E., & Vuori, I. M. Feasibility of a 2-km walking test for fitness assessment in a population study. Scand J Med Sci Sports. 1992;20(2):119-26.

36. Fjørtoft I, Pedersen, A. V., Sigmundsson, H., & Vereijken, B. Measuring physical fitness in children who are 5 to 12 years old with a test battery that is functional and easy to administer. Phys Ther. 2011;91(7):1087-95.

37. Cadenas-Sanchez C, Alcántara-Moral, F., Sanchez-Delgado, G., Mora-Gonzalez, J., Martinez-Tellez, B., Herrador-Colmenero, M., & Ortega, F. B. Assessment of cardiorespiratory fitness in preschool children: adaptation of the 20 metres shuttle run test. Nutr Hosp. 2014;30(6):1333-43.

38. Cadenas-Sanchez C, Martinez-Tellez B, Sanchez-Delgado G, Mora-Gonzalez J, Castro-Piñero J, Löf M, et al. Assessing physical fitness in preschool children: Feasibility, reliability and practical recommendations for the PREFIT battery. J Sci Med Sport. 2016;19(11):910-5.

39. España-Romero V, Artero EG, Jimenez-Pavón D, Cuenca-Garcia M, Ortega FB, Castro-Piñero J, et al. Assessing health-related fitness tests in the school setting: reliability, feasibility and safety; the ALPHA Study. Int J Sports Med. 2010;31(7):490-7.

40. Aadahl M, Zacho, M., Linneberg, A., Thuesen, B. H., & Jørgensen, T. Comparison of the Danish step test and the watt-max test for estimation of maximal oxygen uptake: the Health 2008 study. Eur J Prev Cardiol. 2013;20(6):1088-94.

41. Hébert LJ, Maltais, D. B., Lepage, C., Saulnier, J., Crête, M., & Perron, M. . Isometric muscle strength in youth assessed by hand-held dynamometry: A feasibility, reliability, and validity study: A feasibility, reliability, and validity study. Pediatr Phys Ther. 2011;23(3):289-99.

42. Smits-Engelsman B, Bonney, E., Neto, J. L. C., & Jelsma, D. L. Feasibility and content validity of the PERF-FIT test battery to assess movement skills, agility and power among children in low-resource settings. BMC Public Health. 2020;20(1):1-11

43. Boyer C, Tremblay, M., Saunders, T., McFarlane, A., Borghese, M., Lloyd, M., & Longmuir, P. Feasibility, validity, and reliability of the plank isometric hold as a field-based assessment of torso muscular endurance for children 8–12 years of age. Pediatr Exerc Sci. 2013;25(3):407-22.

44. Ito T, Shirado, O., Suzuki, H., Takahashi, M., Kaneda, K., & Strax, T. E. Lumbar trunk muscle endurance testing: an inexpensive alternative to a machine for evaluation. Arch Phys Med Rehabil. 1996;77(1):75-9.
