# Supplementary material for: Feasibility and Safety of Field-Based Physical Fitness Tests: A Systematic Review
Source: Sports Med Open. 2025 Jan 24;11:8. doi: 10.1186/s40798-024-00799-1 (PMC11759754; doi:10.1186/s40798-024-00799-1)
Supplement: Supplementary file 5 — Supplementary Material 5. [file 40798_2024_799_MOESM5_ESM.docx]

**Supplementary Table S4.** Quality assessment of field-based fitness test safety studies.

| Study | Fitness Component | Field-based fitness test | Number of study subjects | Description of the study population | Statistical analysis | Total score |
| --- | --- | --- | --- | --- | --- | --- |
| Borel et al., 2010^[30]^ | Cardiorespiratory fitness | 6-min step | 1 | 2 | 1 | 4 |
| Amado-Pacheco et al., 2019^[32]^ | Cardiorespiratory fitness | 20-m shuttle run | 2 | 2 | 0 | 4 |
| Bruggeman et al., 2020^[28]^ | Cardiorespiratory fitness | 3-min step | 1 | 2 | 2 | 5 |
| Anderson & Dal Corso, 2016^[29]^ | Cardiorespiratory fitness | Chester step | 1 | 2 | 2 | 5 |
| Anderson & Dal Corso, 2016^[29]^ | Cardiorespiratory fitness | Modified incremental step | 1 | 2 | 2 | 5 |
| Anderson & Dal Corso, 2016^[29]^ | Cardiorespiratory fitness | 6-min walk | 1 | 2 | 2 | 5 |
| Lamoneda et al., 2020^[31]^ | Cardiorespiratory fitness | 20-m shuttle run music | 2 | 1 | 1 | 5 |
| Lamoneda et al., 2020^[31]^ | Cardiorespiratory fitness | 20-m shuttle run | 2 | 1 | 1 | 5 |
| Aadahl et al., 2012^[40]^ | Cardiorespiratory fitness | Danish step | 2 | 2 | 2 | 6 |
| Oja et al., 1991^[34]^ | Cardiorespiratory fitness | 2-km walk | 2 | 2 | 2 | 6 |
| Laukkanen et al., 1992^[35]^ | Cardiorespiratory fitness | 2-km walk | 2 | 2 | 2 | 6 |
| Suni et al., 1998^[20]^ | Cardiorespiratory fitness | 2-km walk | 2 | 2 | 2 | 6 |
| España-Romero et al., 2010^[39]^ | Cardiorespiratory fitness | 20-m shuttle run | 2 | 2 | 2 | 6 |
| Amado-Pacheco et al., 2019^[32]^ | Musculoskeletal fitness | Handgrip | 2 | 2 | 0 | 4 |
| Amado-Pacheco et al., 2019^[32]^ | Musculoskeletal fitness | Standing long jump | 2 | 2 | 0 | 4 |
| Amado-Pacheco et al., 2019^[32]^ | Musculoskeletal fitness | Sit and reach | 2 | 2 | 0 | 4 |
| Bruggeman et al., 2020^[28]^ | Musculoskeletal fitness | 45-s squat | 1 | 2 | 2 | 5 |
| Hébert et al., 2011^[41]^ | Musculoskeletal fitness | Handgrip | 2 | 2 | 1 | 5 |
| Ito et al., 1996^[44]^ | Musculoskeletal fitness | Trunk flexor endurance | 2 | 2 | 1 | 5 |
| Ito et al., 1996^[44]^ | Musculoskeletal fitness | Isometric back endurance | 2 | 2 | 1 | 5 |
| Smits-Engelsman et al., 2020^[42]^ | Musculoskeletal fitness | Standing long jump | 2 | 1 | 2 | 5 |
| España-Romero et al., 2010^[39]^ | Musculoskeletal fitness | Handgrip | 2 | 2 | 2 | 6 |
| Suni et al., 1998^[20]^ | Musculoskeletal fitness | Handgrip | 2 | 2 | 2 | 6 |
| España-Romero et al., 2010^[39]^ | Musculoskeletal fitness | Standing long jump | 2 | 2 | 2 | 6 |
| Suni et al., 1998^[20]^ | Musculoskeletal fitness | Vertical jump | 2 | 2 | 2 | 6 |
| Suni et al., 1998^[20]^ | Musculoskeletal fitness | Isometric back endurance | 2 | 2 | 2 | 6 |
| Suni et al., 1998^[20]^ | Musculoskeletal fitness | Modified push-ups | 2 | 2 | 2 | 6 |
| Amado-Pacheco et al., 2019^[32]^ | Motor fitness | 4 x 10-m shuttle run | 2 | 2 | 0 | 4 |
| Smits-Engelsman et al., 2020^[42]^ | Motor fitness | Dynamic balance | 2 | 1 | 2 | 5 |
| Smits-Engelsman et al., 2020^[42]^ | Motor fitness | Single-leg stand | 2 | 1 | 2 | 5 |
| Suni et al., 1998^[20]^ | Motor fitness | Single-leg stand | 2 | 2 | 2 | 6 |

Total score indicates high quality = 5-6; low quality = 3-4; very low quality = 0-2.

**REFERENCES**

20. Suni JH, Miilunpalo, S. I., Asikainen, T. M., Laukkanen, R. T., Oja, P., Pasanen, M. E., & Vuori, I. M. Safety and feasibility of a health-related fitness test battery for adults. Phys Ther. 1998;78(2):134-48.

28. Bruggeman BS, Vincent, H. K., Chi, X., Filipp, S. L., Mercado, R., Modave, F., & Bernier, A. Simple tests of cardiorespiratory fitness in a pediatric population. Plos one. 2020;15(9).

29. José A, & Dal Corso, S. Step tests are safe for assessing functional capacity in patients hospitalized with acute lung diseases. J Cardiopulm Rehabil Prev. 2016;36(1):56-61.

30. Borel B, Fabre, C., Saison, S., Bart, F., & Grosbois, J. M. An original field evaluation test for chronic obstructive pulmonary disease population: the six-minute stepper test. Clin Rehabil. 2010;24(1):82-93.

31. Lamoneda J, Huertas-Delgado, F. J., & Cadenas-Sanchez, C. Feasibility and concurrent validity of a cardiorespiratory fitness test based on the adaptation of the original 20 m shuttle run: The 20 m shuttle run with music. J Sports Sci. 2021;39(1):57-63.

32. Amado-Pacheco JC, Prieto-Benavides DH, Correa-Bautista JE, García-Hermoso A, Agostinis-Sobrinho C, María Alonso-Martínez A., et al. Feasibility and reliability of physical fitness tests among colombian preschool children. Int J Environ Res Public Health. 2019;16(17):3069.

34. Oja P, Laukkanen, R., Pasanen, M., Tyry, T., & Vuori, I. A 2-km walking test for assessing the cardiorespiratory fitness of healthy adults. Int J Sports Med. 1991;12(4):356-62.

35. Laukkanen RM, Oja, P., Ojala, K. H., Pasanen, M. E., & Vuori, I. M. Feasibility of a 2-km walking test for fitness assessment in a population study. Scand J Med Sci Sports. 1992;20(2):119-26.

39. España-Romero V, Artero EG, Jimenez-Pavón D, Cuenca-Garcia M, Ortega FB, Castro-Piñero J, et al. Assessing health-related fitness tests in the school setting: reliability, feasibility and safety; the ALPHA Study. Int J Sports Med. 2010;31(7):490-7.

40. Aadahl M, Zacho, M., Linneberg, A., Thuesen, B. H., & Jørgensen, T. Comparison of the Danish step test and the watt-max test for estimation of maximal oxygen uptake: the Health 2008 study. Eur J Prev Cardiol. 2013;20(6):1088-94.

41. Hébert LJ, Maltais, D. B., Lepage, C., Saulnier, J., Crête, M., & Perron, M. . Isometric muscle strength in youth assessed by hand-held dynamometry: A feasibility, reliability, and validity study: A feasibility, reliability, and validity study. Pediatr Phys Ther. 2011;23(3):289-99.

42. Smits-Engelsman B, Bonney, E., Neto, J. L. C., & Jelsma, D. L. Feasibility and content validity of the PERF-FIT test battery to assess movement skills, agility and power among children in low-resource settings. BMC Public Health. 2020;20(1):1-11.

44. Ito T, Shirado, O., Suzuki, H., Takahashi, M., Kaneda, K., & Strax, T. E. Lumbar trunk muscle endurance testing: an inexpensive alternative to a machine for evaluation. Arch Phys Med Rehabil. 1996;77(1):75-9.
