# Supplementary material for: Feasibility and Safety of Field-Based Physical Fitness Tests: A Systematic Review
Source: Sports Med Open. 2025 Jan 24;11:8. doi: 10.1186/s40798-024-00799-1 (PMC11759754; doi:10.1186/s40798-024-00799-1)
Supplement: Supplementary file 6 — Supplementary Material 6. [file 40798_2024_799_MOESM6_ESM.docx]

**Supplementary Table S5.** Overview of field-based fitness test feasibility studies by study quality.

| **Author** | **Participants** | **Age, range/ SD** | **Field-based fitness test** | **Feasibility items** | **Results** | **Conclusions** |
| --- | --- | --- | --- | --- | --- | --- |
| ***Very low quality studies*** |  |  |  |  |  |  |
| *Musculoskeletal fitness (endurance)* |  |  |  |  |  |  |
| McAllister & Palombaro, 2019^[27]^ | 33 elderly  Healthy | 85.2 ± 8.0 | Modified 30-s sit-to-stand | - Participants performing at least 1 repetition of the test (%)  - Time to perform the test (minutes) | 82% of the participants perform at least 1 repetition of the test.  30 seconds. | Modified 30-s sit-to-stand was considered feasible. |
| ***Low quality studies*** |  |  |  |  |  |  |
| *Cardiorespiratory fitness* |  |  |  |  |  |  |
| Bruggeman et al., 2020^[28]^ | 40 Children  Males (n = 23)  Females (n = 17)  Healthy | 12.8 ± 1.9  13.3 ± 1.7  12.1 ± 2.0 | 3-min step | - Time to perform the test (minutes)  - Self-perception effort (%)  - Easy to administer | 3 minutes.  100% of the participants found the test as “low effort”.  Test was considered easy to administer. | 3-min step was considered feasible. |
| Anderson & Dal Corso, 2016^[29]^ | 20 adults  Healthy | 52  41 - 66 | Chester step | - Participants performing the test (%)  - Time to perform the test (minutes) | 100% of the participants perform the test.  10 minutes. | Chester step was considered feasible. |
|  |  |  | Modified incremental step |  | 100% of the participants perform the test.  12 minutes. | Modified incremental step was considered feasible. |
| Borel et al., 2010^[30]^ | 15 adults  Healthy | 29.0 ± 6.0 | 6-min step | - Participants performing the test (%)  - Time to perform the test (minutes) | 100% of the participants perform the test.  6 minutes. | 6-min step was considered feasible. |
| Anderson & Dal Corso, 2016^[29]^ | 20 adults  Healthy | 52  41 - 66 | 6-min walk | - Participants performing the test (%)  - Time to perform the test (minutes) | 100% of the participants perform the test.  6 minutes. | 6-min walk seems to be feasible. |
| Lamoneda et al., 2020^[31]^ | 386 adolescents  Males (n = 189)  Females (n = 197)  Healthy | 14.5 ± 1.6  14.4 ± 1.6  14.5 ± 1.6 | 20-m shuttle run music  vs  20-m shuttle run | - Self-reported questionnaire items by participants: “preference”, “enjoyment”, “maximum effort” and “perception of fatigue” (%) | Preference = 89.6% vs. 10.4%.  Enjoyment = 94% vs. 6%.  Maximum effort = 72.4 both tests.  Less fatigue perception: 56.4% for 20-m shuttle run music. | 20-m shuttle run music and 20-m shuttle run seem to be feasible and 20-m shuttle run music seems to be more feasible. |
| *Musculoskeletal fitness (endurance)* |  |  |  |  |  |  |
| Bruggeman et al., 2020^[28]^ | 40 Children  Males (n = 23)  Females (n = 17)  Healthy | 12.8 ± 1.9  13.3 ± 1.7  12.1 ± 2.0 | 45-s squat | - Time to perform the test (minutes)  - Self-perception effort (%)  - Easy to administer | 45 seconds.  100% of the participants found the test as “low effort”.  Test was considered easy to administer. | 45-s squat was considered feasible. |
| ***High quality studies*** |  |  |  |  |  |  |
| *Cardiorespiratory fitness* |  |  |  |  |  |  |
| Aadahl et al., 2012^[40]^ | 749 adults  Males (n=346)  Females (n=449)  Healthy | 46.8 ± 8.2  46.7 ± 8.4  47.0 ± 8.0 | Danish step | - Participants performing the test (%)  - Time to perform the test (minutes) | 98.8% of the participants perform the test.  6 minutes. | Danish step was considered feasible. |
| Langhammer & Stanghelle, 2018^[33]^ | 172 elderly  Healthy | 73 ± 5.8 | 2-min step | - Participants performing the test (%)  - Time to perform the test (minutes) | 100% of the participants perform the test.  2 minutes. | 2-min step was considered feasible. |
|  |  |  | 6-min walk |  | 100% of the participants perform the test.  6 minutes. | 6-min walk seems to be feasible. |
| Oja et al., 1991^[34]^ | 64 adults  Males (n=35)  Females (n=29)  Healthy | 41 ± 13.7  42.9 ± 14.0  39.1 ± 13.4 | 2-km walk | - Participants performing the test (%)  - Time to perform the test (minutes, mean (SD)) | 100% of the participants perform the test.  Males = 15.2 (1.8) minutes  Females = 16.9 (1.5) minutes. | 2-km walk seems to be feasible. |
| Laukkanen et al., 1992^[35]^ | 273 adults  Males (n = 121)  Females (n = 152)  Healthy | 44.5 ± 10.3  44.5 ± 10.2  44.5 ± 10.5 | 2-km walk | - Participants performing the test (%)  - Time to perform the test (minutes)  - Reject to perform the test (%)  - Number of evaluators (n)  - Self-perception effort (%) | 96.4% of the participants perform the test.  15-18 minutes.  21% of the participants rejected to perform the test.  2 evaluators.  81% of the participants found the test “easy”, 17% “somewhat difficult” and 2% “difficult”. | 2-km walk seems to be feasible. |
| Suni et al., 1998^[20]^ | 600 adults  Males (n = 246)  Females (n = 354)  Healthy | 37 - 57  47.0 ± 7.9  47.0 ± 8.6 | 2-km walk | - Participants performing the test (%)  - Time to perform the test (minutes)  - Number of evaluators (n)  - Easy to administer | 95% of the participants performed the walk test.  20-25 minutes.  3 evaluators.  Test was considered easy to administer. | 2-km walk seems to be feasible. |
| Amado-Pacheco et al., 2019^[32]^ | 90 children  Males (n = 48)  Females (n = 42)  Healthy | 4.04 ± 0.82  3.95 ± 0.82 | 20-m shuttle run | - Participants performing the test (%)  - Time to perform the test (minutes, mean (SD)) | 96% of the participants perform the test.  Time males = 6.7 (3).  Time females = 5.3 (2). | 20-m shuttle run seems to be feasible. |
| Cadenas-Sanchez et al., 2014^[37]^ | 137 children  Males (n = 78)  Females (n =59)  Healthy | 4.91 ± 0.89  4.9 ± 0.8  4.8 ± 0.8 | 20-m shuttle run | - Time to perform the test (minutes)  - Time to prepare the test (minutes)  - Participants per group (n)  - Number of evaluators (n) | 8-10 minutes (including explanations and demonstrations).  3 minutes.  Groups of 4-8 participants, 4-5 participants if they are 3-year-old.  A minimum of 2 evaluators running with children. | 20-m shuttle run seems to be feasible. |
| Cadenas-Sánchez et al., 2016^[38]^ | 161 children  Males (n = 92)  Females (n = 69)  Healthy | 4.91 ± 0.83  4.94 ± 0.86  4.87 ± 0.79 | 20-m shuttle run | - Time to perform the test (minutes)  - Participants per group (n)  - Number of evaluators (n)  - Understanding the test instruction (%)  - Easy to administer | 8-10 minutes (including explanations and demonstrations).  Groups of 4-8 participants of the same age.  A minimum of 2 evaluators running with children.  100% of the participants understood the test.  Test was considered easy to administer. | 20-m shuttle run seems to be feasible. |
| España-Romero et al., 2010^[39]^ | 58 children  80 adolescents  Healthy | 6 - 11.9  12 - 18 | 20-m shuttle run | - Time to prepare the test (minutes)  - Time to perform the test (minutes)  - Participants per group (n)  - Number of evaluators (n)  - Questionnaire with items of dichotomous answer (%)  - Easy to administer | ~5 minutes of preparation.  ~10 minutes of perform.  20 participants.  1 evaluator (Physical Education teachers at school).  99.2% of participants wore appropriate sport clothes, 100% understood test instructions and 0.8% rejected to perform the test by lack of motivation. 100% facilities were appropriate.  Test was considered easy to administer. | 20-m shuttle run seems to be feasible. |
| Fjortoft et al., 2011^[36]^ | 195 children  Males (n = 94)  Females (n = 101)  Healthy | 5-12 | 6-min run | - Participants performing the test (%)  - Understanding the test instruction (%)  - Easy to administer | 100% of the participants perform the test.  100% of the participants understood the test.  Test was considered easy to administer. | 6-min run was considered feasible. |
| *Musculoskeletal fitness (isometric)* |  |  |  |  |  |  |
| Hébert et al., 2011^[41]^ | 33 children  41 adolescents  Males (n = 37)  Females (n = 37)  Healthy | 4 - 9.9  10 - 17.5 | Handgrip | - Understanding the test instruction (%)  - Easy to administer | 100% of the participants understood the test.  Test was considered easy to administer. | Handgrip was considered feasible. |
| Amado-Pacheco et al., 2019^[32]^ | 90 children  Males (n = 48)  Females (n = 42)  Healthy | 4.04 ± 0.82  3.95 ± 0.82 | Handgrip | - Participants performing the test (%) | 100% of the participants performed the test. | Handgrip was considered feasible. |
| Cadenas-Sánchez et al., 2016^[38]^ | 161 children  Males (n = 92)  Females (n = 69)  Healthy | 4.91 ± 0.83  4.94 ± 0.86  4.87 ± 0.79 | Handgrip | - Time to perform the test (minutes)  - Participants per group (n)  - Understanding the test instruction (%)  - Easy to administer | 8-10 minutes (including explanations and demonstrations)  Groups of maximum 8 participants.  100% of the participants understood the test.  Test was considered easy to administer. | Handgrip was considered feasible. |
| España-Romero et al., 2010^[39]^ | 58 children  80 adolescents  Healthy | 6 - 11.9  12 - 18 | Handgrip | - Time to prepare the test (minutes)  - Time to perform the test (minutes)  - Participants per group (n)  - Number of evaluators (n)  - Questionnaire with items of dichotomous answer (%)  - Easy to administer | ~3 minutes of preparation.  ~90 seconds of perform by participants.  Groups of 20 participants.  1 evaluator (Physical Education  teachers at school).  99.2% of participants wore appropriate sport clothes, 98.5% understood test instructions and 0% rejected to perform the test by lack of motivation. 100% facilities were appropriate.  Test was considered easy to administer. | Handgrip was considered feasible. |
| Suni et al., 1998^[20]^ | 600 adults  Males (n = 246)  Females (n = 354)  Healthy | 37 - 57  47.0 ± 7.9  47.0 ± 8.6 | Handgrip | - Time to prepare and perform the test (5 points scale (mean))  - Participants performing the test (%)  - Easy to administer | Mean score = 4.3-4.7 (excellent).  95% of the participants perform the test.  Test was considered easy to administer. | Handgrip was considered feasible. |
| *Musculoskeletal fitness (explosive)* |  |  |  |  |  |  |
| Fjortoft et al., 2011^[36]^ | 195 children  Males (n = 94)  Females (n = 101)  Healthy | 5-12 | Standing long jump | - Participants performing the test (%)  - Acceptance of the test (%)  - Easy to administer | 100% of the participants perform the test.  100% of the participants shows acceptance for the test.  Test was considered easy to administer. | Standing long jump was considered feasible. |
| Amado-Pacheco et al., 2019^[32]^ | 90 children  Males (n = 48)  Females (n = 42)  Healthy | 4.04 ± 0.82  3.95 ± 0.82 | Standing long jump | - Participants performing the test (%) | 100% of the participants performed the test. | Standing long jump was considered feasible. |
| Cadenas-Sánchez et al., 2016^[38]^ | 161 children  Males (n = 92)  Females (n = 69)  Healthy | 4.91 ± 0.83  4.94 ± 0.86  4.87 ± 0.79 | Standing long jump | - Time to perform the test (minutes)  - Participants per group (n)  - Understanding the test instruction (%)  - Easy to administer | 8-10 minutes (including explanations and demonstrations)  Groups of maximum 8 participants.  100% of the participants understood the test.  Test was considered difficult to administer (several more trials are required). | Standing long jump was considered feasible; however, it was needed more trials to perform it correctly (because it requires plenty of coordination). |
| Smits-Engelsman et al., 2020^[42]^ | 80 children  Males (n = 39)  Females (n = 41)  Healthy | 9.2 ± 1.1 | Standing long jump | - Participants performing the test (%)  - Understanding the test instruction (%)  - Appropriate sport clothes (%)  - Easy to administer  - Space (m) | 100% of the participants performed the test.  100% of the participants understood the test.  76% appropriate sport clothes.  Test was considered easy to administer.  5–6m long by 3–4m wide. | Standing long jump seems to be feasible. |
| España-Romero et al., 2010^[39]^ | 58 children  80 adolescents  Healthy | 6 - 11.9  12 - 18 | Standing long jump | - Time to prepare the test (minutes)  - Time to perform the test (minutes)  - Participants per group (n)  - Number of evaluators (n)  - Questionnaire with items of dichotomous answer (%)  - Easy to administer | ~3 minutes of preparation.  ~50 seconds of perform by participants.  Groups of 20 participants.  1 evaluator (Physical Education teachers at school).  99.2% of participants wore appropriate sport clothes, 99.2% understood test instructions and 0.8% rejected to perform the test by lack of motivation. 100% facilities were appropriate.  Test was considered easy to administer. | Standing long jump seems to be feasible. |
| Suni et al., 1998^[20]^ | 600 adults  Males (n = 246)  Females (n = 354)  Healthy | 37 - 57  47.0 ± 7.9 | Vertical jump | - Time to prepare and perform the test (5 points scale (mean score))  - Participants performing the test (%)  - Easy to administer | Mean score = 5.0 (excellent).  95% of the participants perform the test.  Test was considered easy to administer. | Standing long jump was considered feasible. |
| Fjortoft et al., 2011^[36]^ | 195 children  Males (n = 94)  Females (n = 101)  Healthy | 5-12 | Medicine ball push (1 kg) | - Participants performing the test (%)  - Understanding the test instruction (%)  - Easy to administer | 100% of the participants perform the test.  100% of the participants understood the test.  Test was considered easy to administer. | Medicine ball push (1 kg) seems to be feasible. |
| *Musculoskeletal fitness (endurance)* |  |  |  |  |  |  |
| Langhammer & Stanghelle, 2018^[33]^ | 172 elderly  Healthy | 73 ± 5.8 | 30-s sit-to-stand | - Participants performing the test (%)  -Time to perform the test (minutes) | 100% of the participants perform the test.  30 seconds. | 30-s sit-to-stand was considered feasible. |
|  |  |  | Arm curl |  | 100% of the participants perform the test. | Arm curl was considered feasible. |
| Boyer et al., 2013^[43]^ | 823 children  Males (n = 395)  Females (n = 428)  Healthy | 8-12 | Fitnessgram partial curl-ups  CHMS (Canadian Health Measures Surve) partial curl-ups  60-s plank  90-s plank  Unlimited time plank | - Participants performing the test (%): zero (floor effect), interpretable (scores between nonzero and a nonmaximum), and maximum scores (ceiling effect].  - Time to perform the tests (seconds, mean (SD)) | 12%, 5% and 0.8% of participants cannot perform (zero score) the partial curl-up (Fitnessgram and CHMS) and plank protocols tests, respectively.  CHMS partial curl-ups: Only differentiates 70% of participants.  60-s plank: only differentiates 55% of participants.  90-s plank: only differentiates 86% of participants.  Unlimited time plank:133s was required to provide a nonzero/ nonmaximal score for 95% of the participants.  Fitnessgram partial curl-ups (29 s (28)), CHMS partial curl-ups (36 s (21)), 60-s plank (41 s (16)), 90-s plank (52 s (25)) and unlimited time (56 s (40)). | The unlimited plank was considered feasible (it takes significantly more time to administer in comparison with the Fitnessgram partial curl-ups protocol). |
| Ito et al., 1996^[44]^ | 90 adults  Males (n = 37)  Females (n = 53)  Healthy | 44.3  46.8 | Trunk flexor endurance  Isometric back endurance | -Participants performing the test (%)  - Time to perform the test (minutes, mean (SD))  - Easy to administer | 100 % of the participants perform the test.  Male = 182.6 (69.3) minutes.  Female = 208.2 (66.2) minutes.  4.4% participants were able to achieve more than 5 minutes.  Test was considered easy to administer.  100 % of the participants perform the test.  Male = 85.1 (44.8) minutes.  Female = 128.4 (53.0) minutes.  11.1% participants were able to achieve more than 5 minutes.  Test was considered easy to administer. | Trunk flexor and isometric back endurance tests were considered feasible. |
| Suni et al., 1998^[20]^ | 600 adults  Males (n = 246)  Females (n = 354)  Healthy | 37 - 57  47.0 ± 7.9 | Isometric back endurance  Modified push-ups | - Participants performing the test (%)  - Time to prepare and perform the test (5 points scale (mean score))  - Easy to administer | 95% of the participants perform the test.  Mean score = 4.3-4.7 (excellent).  Test was considered easy to administer.  95% of the participants perform the test.  Mean score = 4.3-4.7 (excellent).  Test was considered easy to administer. | Isometric back endurance was considered feasible.  Modified push-ups was considered feasible. |
| *Musculoskeletal fitness (flexibility)* |  |  |  |  |  |  |
| Amado-Pacheco et al., 2019^[32]^ | 90 children  Males (n = 48)  Females (n = 42)  Healthy | 4.04 ± 0.82  3.95 ± 0.82 | Sit and reach | - Participants performing the test (%) | 100% of the participants performed the test. | Sit and reach and was considered feasible. |
| Langhammer & Stanghelle, 2018^[33]^ | 172 elderly  Healthy | 73 ± 5.8 | Chair sit and reach | - Participants performing the test (%) | 100% of the participants perform the test. | Chair sit and reach was considered feasible. |
|  |  |  | Back scratch |  | 100% of the participants perform the test. | Back scratch was considered feasible. |
| *Motor fitness (speed)* |  |  |  |  |  |  |
| Fjortoft et al., 2011^[36]^ | 195 children  Males (n = 94)  Females (n = 101)  Healthy | 5-12 | 20-m run | - Participants performing the test (%)  - Understanding the test instruction (%)  - Easy to administer | 100% of the participants perform the test.  100% of the participants understood the test.  Test was considered easy to administer. | 20-m run seems to be feasible. |
| *Motor fitness (agility)* |  |  |  |  |  |  |
| Amado-Pacheco et al., 2019^[32]^ | 90 children  Males (n = 48)  Females (n = 42)  Healthy | 4.04 ± 0.82  3.95 ± 0.82 | 4 × 10-m shuttle run | - Participants performing the test (%)  - Time to perform the test (seconds, mean (SD)) | 100% of the participants performed the test.  Time males = 17.3 (2.5)  Time females = 18.7 (3.1). | 4 × 10-m shuttle run was considered feasible. |
| Cadenas-Sánchez et al., 2016^[38]^ | 161 children  Males (n = 92)  Females (n = 69)  Healthy | 4.91 ± 0.83  4.94 ± 0.86  4.87 ± 0.79 | 4 × 10-m shuttle run | - Time to perform the test (minutes)  - Participants per group (n)  - Number of evaluators (n)  - Understanding the test instruction (%)  - Easy to administer | 8-10 minutes (including explanations and demonstrations)  Groups of maximum 8 participants.  2 evaluators were positioned in both extremes and participants had to touch the evaluator hand.  100% of the participants understood the test.  Test was considered easy to administer. | 4 × 10-m shuttle run was considered feasible. |
| Fjortoft et al., 2011^[36]^ | 195 children  Males (n = 94)  Females (n = 101)  Healthy | 5-12 | 10 × 5-m shuttle run | - Participants performing the test (%)  - Understanding the test instruction (%)  - Easy to administer | 100% of the participants perform the test.  100% of the participants understood the test.  Test was considered easy to administer. | 10 × 5-m shuttle run was considered feasible. |
| Langhammer & Stanghelle, 2018^[33]^ | 172 elderly  Healthy | 73 ± 5.8 | 2.45-m time up & go | - Participants performing the test (%) | 100% of the participants perform the test. | 2.45-m up and go was considered feasible. |
| *Motor fitness (balance)* |  |  |  |  |  |  |
| Cadenas-Sánchez et al., 2016^[38]^ | 161 children  Males (n = 92)  Females (n = 69)  Healthy | 4.91 ± 0.83  4.94 ± 0.86  4.87 ± 0.79 | Single-leg stand | - Time to perform the test (minutes)  - Subjects per group (n)  - Understanding the test instruction (%)  - Easy to administer | 8-10 minutes (including explanations and demonstrations)  Groups of maximum 8 participants.  100% of the participants understood the test.  Test was considered easy to administer. | Single-leg stand was considered feasible. |
| Smits-Engelsman et al., 2020^[42]^ | 80 children  Males (n = 39)  Females (n = 41)  Healthy | 9.2 ± 1.1 | Singe-leg stand  Dynamic balance | - Participants performing the test (%)  - Understanding the test instruction (%)  - Appropriate sport clothes (%)  - Easy to administer  - Space (m) | 100% of the participants performed the test.  100% of the participants understood the test.  76% of participants wore appropriate sport clothes.  Test was considered easy to administer.  5–6m long by 3–4m wide.  100% of the participants performed the test.  100% of the participants understood the test.  76% of the participants wore appropriate sport clothes.  Test was considered easy to administer.  5–6m long by 3–4m wide. | Singe-leg stand and dynamic balance were considered feasible. |
| Suni et al., 1998^[20]^ | 600 adults  Males (n = 246)  Females (n = 354)  Healthy | 37 - 57  47.0 ± 7.9 | Single-leg stand | - Time to prepare and perform the test (5 points scale (mean score))  - Participants performing the test (%)  - Easy to administer | Mean score = 4.3-4.7 (excellent).  95% of the participants perform the test.  Test was considered easy to administer. | Single-leg stand was considered feasible. |
| *Motor fitness (multi-skills)* |  |  |  |  |  |  |
| Fjortoft et al., 2011^[36]^ | 195 children  Males (n = 94)  Females (n = 101)  Healthy | 5-12 | Jumping a distance of 7-m on 1 foot | - Performance of the test (%)  - Understanding the test instruction (%)  - Easy to administer | 100% of the participants perform the test.  100% of the participants understood the test.  Test was considered easy to administer. | Jumping a distance of 7-m on 1 foot, seems to be feasible. |
| Fjortoft et al., 2011^[36]^ | 195 children  Males (n = 94)  Females (n = 101)  Healthy | 5-12 | Jumping a distance of 7-m on 2 feet | - Performance of the test (%)  - Understanding the test instruction (%)  - Easy to administer | 100% of the participants perform the test.  100% of the participants understood the test.  Test was considered easy to administer. | Jumping a distance of 7-m on 2 feet, seems to be feasible. |

**REFERENCES**

20. Suni JH, Miilunpalo, S. I., Asikainen, T. M., Laukkanen, R. T., Oja, P., Pasanen, M. E., & Vuori, I. M. Safety and feasibility of a health-related fitness test battery for adults. Phys Ther. 1998;78(2):134-48.

27. McAllister LS, & Palombaro, K. M. Modified 30-second sit-to-stand test: reliability and validity in older adults unable to complete traditional sit-to-stand testing. J Geriatr Phys Ther. 2020;43(3):153-8.

28. Bruggeman BS, Vincent, H. K., Chi, X., Filipp, S. L., Mercado, R., Modave, F., & Bernier, A. Simple tests of cardiorespiratory fitness in a pediatric population. Plos one. 2020;15(9).

29. José A, & Dal Corso, S. Step tests are safe for assessing functional capacity in patients hospitalized with acute lung diseases. J Cardiopulm Rehabil Prev. 2016;36(1):56-61.

30. Borel B, Fabre, C., Saison, S., Bart, F., & Grosbois, J. M. An original field evaluation test for chronic obstructive pulmonary disease population: the six-minute stepper test. Clin Rehabil. 2010;24(1):82-93.

31. Lamoneda J, Huertas-Delgado, F. J., & Cadenas-Sanchez, C. Feasibility and concurrent validity of a cardiorespiratory fitness test based on the adaptation of the original 20 m shuttle run: The 20 m shuttle run with music. J Sports Sci. 2021;39(1):57-63.

32. Amado-Pacheco JC, Prieto-Benavides DH, Correa-Bautista JE, García-Hermoso A, Agostinis-Sobrinho C, María Alonso-Martínez A., et al. Feasibility and reliability of physical fitness tests among colombian preschool children. Int J Environ Res Public Health. 2019;16(17):3069.

33. Langhammer B, & Stanghelle, J. K. Senior fitness test; a useful tool to measure physical fitness in persons with acquired brain injury. Brain Inj. 2019;33(2):183-8.

34. Oja P, Laukkanen, R., Pasanen, M., Tyry, T., & Vuori, I. A 2-km walking test for assessing the cardiorespiratory fitness of healthy adults. Int J Sports Med. 1991;12(4):356-62.

35. Laukkanen RM, Oja, P., Ojala, K. H., Pasanen, M. E., & Vuori, I. M. Feasibility of a 2-km walking test for fitness assessment in a population study. Scand J Med Sci Sports. 1992;20(2):119-26.

36. Fjørtoft I, Pedersen, A. V., Sigmundsson, H., & Vereijken, B. Measuring physical fitness in children who are 5 to 12 years old with a test battery that is functional and easy to administer. Phys Ther. 2011;91(7):1087-95.

37. Cadenas-Sanchez C, Alcántara-Moral, F., Sanchez-Delgado, G., Mora-Gonzalez, J., Martinez-Tellez, B., Herrador-Colmenero, M., & Ortega, F. B. Assessment of cardiorespiratory fitness in preschool children: adaptation of the 20 metres shuttle run test. Nutr Hosp. 2014;30(6):1333-43.

38. Cadenas-Sanchez C, Martinez-Tellez B, Sanchez-Delgado G, Mora-Gonzalez J, Castro-Piñero J, Löf M, et al. Assessing physical fitness in preschool children: Feasibility, reliability and practical recommendations for the PREFIT battery. J Sci Med Sport. 2016;19(11):910-5.

39. España-Romero V, Artero EG, Jimenez-Pavón D, Cuenca-Garcia M, Ortega FB, Castro-Piñero J, et al. Assessing health-related fitness tests in the school setting: reliability, feasibility and safety; the ALPHA Study. Int J Sports Med. 2010;31(7):490-7.

40. Aadahl M, Zacho, M., Linneberg, A., Thuesen, B. H., & Jørgensen, T. Comparison of the Danish step test and the watt-max test for estimation of maximal oxygen uptake: the Health 2008 study. Eur J Prev Cardiol. 2013;20(6):1088-94.

41. Hébert LJ, Maltais, D. B., Lepage, C., Saulnier, J., Crête, M., & Perron, M. . Isometric muscle strength in youth assessed by hand-held dynamometry: A feasibility, reliability, and validity study: A feasibility, reliability, and validity study. Pediatr Phys Ther. 2011;23(3):289-99.

42. Smits-Engelsman B, Bonney, E., Neto, J. L. C., & Jelsma, D. L. Feasibility and content validity of the PERF-FIT test battery to assess movement skills, agility and power among children in low-resource settings. BMC Public Health. 2020;20(1):1-11

43. Boyer C, Tremblay, M., Saunders, T., McFarlane, A., Borghese, M., Lloyd, M., & Longmuir, P. Feasibility, validity, and reliability of the plank isometric hold as a field-based assessment of torso muscular endurance for children 8–12 years of age. Pediatr Exerc Sci. 2013;25(3):407-22.

44. Ito T, Shirado, O., Suzuki, H., Takahashi, M., Kaneda, K., & Strax, T. E. Lumbar trunk muscle endurance testing: an inexpensive alternative to a machine for evaluation. Arch Phys Med Rehabil. 1996;77(1):75-9.
