# Supplementary material for: Feasibility and Safety of Field-Based Physical Fitness Tests: A Systematic Review
Source: Sports Med Open. 2025 Jan 24;11:8. doi: 10.1186/s40798-024-00799-1 (PMC11759754; doi:10.1186/s40798-024-00799-1)
Supplement: Supplementary file 7 — Supplementary Material 7. [file 40798_2024_799_MOESM7_ESM.docx]

**Supplementary Table S6.** Overview of field-based fitness test safety studies by study quality.

| **Author** | **Participants** | **Age, range/ SD** | **Field-based fitness test** | **Safety items** | **Results** | **Conclusions** |
| --- | --- | --- | --- | --- | --- | --- |
| ***Low quality studies*** |  |  |  |  |  |  |
| *Cardiorespiratory fitness* |  |  |  |  |  |  |
| Borel et al., 2010^[30]^ | 15 adults  Healthy | 29.0 ± 6.0 | 6-min step | -Heart rate ((HR), bpm) (mean (SD))  -Dyspnea  (Borg scale 0-10) (mean (SD))  -Leg fatigue  (Borg scale 0-10)  (mean (SD)) | HR = 118.2 (18.7).  Borg scale dyspnea = 2.5 (1.5).  Borg scale leg fatigue = 3.4 (2.5). | 6-min step was considered safe. |
| Amado-Pacheco et al., 2019^[32]^ | 90 children  Males (n = 48)  Females (n = 42)  Healthy | 4.04 ± 0.82  3.95 ± 0.82 | 20-m shuttle run | -Adverse events (%) | 100% of participants perform the test without adverse events. | 20-m shuttle run seems to be safe. |
| *Musculoskeletal fitness (isometric)* |  |  |  |  |  |  |
| Amado-Pacheco et al., 2019^[32]^ | 90 children  Males (n = 48)  Females (n = 42)  Healthy | 4.04 ± 0.82  3.95 ± 0.82 | Handgrip | -Adverse events (%) | 100% of participants perform the test without adverse events. | Handgrip was considered safe. |
| *Musculoskeletal fitness (explosive)* |  |  |  |  |  |  |
| Amado-Pacheco et al., 2019^[32]^ | 90 children  Males (n = 48)  Females (n = 42)  Healthy | 4.04 ± 0.82  3.95 ± 0.82 | Standing long jump | -Adverse events (%) | 100% of participants perform the test without adverse events. | Standing long jump seems to be safe. |
| *Musculoskeletal fitness (flexibility)* |  |  |  |  |  |  |
| Amado-Pacheco et al., 2019^[32]^ | 90 children  Males (n = 48)  Females (n = 42)  Healthy | 4.04 ± 0.82  3.95 ± 0.82 | Sit and reach | -Adverse events (%) | 100% of participants perform the test without adverse events. | Sit and reach was considered safe. |
| *Motor fitness (agility)* |  |  |  |  |  |  |
| Amado-Pacheco et al., 2019^[32]^ | 90 children  Males (n = 48)  Females (n = 42)  Healthy | 4.04 ± 0.82  3.95 ± 0.82 | 4 × 10-m shuttle run | -Adverse events (%) | 100% of participants perform the test without adverse events. | 4 × 10-m shuttle run was considered safe. |
| ***High quality studies*** |  |  |  |  |  |  |
| *Cardiorespiratory fitness* |  |  |  |  |  |  |
| Bruggeman et al., 2020^[28]^ | 40 Children  Males (n = 23)  Females (n = 17)  Healthy | 12.8 ± 1.9  13.3 ± 1.7  12.1 ± 2.0 | 3-min step | -Rating of perceived exertion (RPE) (Borg scale 0-10) (mean (SD))  -HR (bpm) (mean (SD))  -Adverse events (%) | RPE = 2.7 (1.4).  HR not available data.  100% of participants perform the test without adverse events. | 3-min step was considered safe. |
| Anderson & Dal Corso, 2016^[29]^ | 20 adults  Healthy | 52  41 - 66 | Chester step | -HR (bpm) (mean (SD))  -Dyspnea (Borg scale 0-10) (mean (range))  -Leg fatigue (Borg scale 0-10) (mean (range))  -Adverse events (%) | HR = 143 (28.0).  Borg scale dyspnea = 2 (1-3).  Borg scale leg fatigue = 2 (1-3).  100% of participants perform the test without adverse events. | Chester step was considered safe. |
|  |  |  | Modified incremental step |  | HR = 142 (27.0).  Borg scale dyspnea = 2 (1-3).  Borg scale leg fatigue = 2 (1-4).  100% of participants perform the test without adverse events. | Modified incremental step was considered safe. |
| Aadahl et al., 2012^[40]^ | 795 adults  Males (n=346)  Females (n=449)  Healthy | 46.8 ± 8.2  46.7 ± 8.4  47.0 ± 8.0 | Danish step | -Adverse events (n) | 1 participant had a strained calf muscle after 30 sec, the test was not finished. | Danish step was considered safe. |
| Suni et al., 1998^[20]^ | 600 adults  Males (n = 246)  Females (n = 354)  Healthy | 37 - 57  47.0 ± 7.9  47.0 ± 8.6 | 2-km walk | -Delayed-onset muscle soreness (DOMS) questionnaire (%)  -HR (bpm) (mean (SD))  -Exertion 85%HRmax (%)  -Adverse events (%) | DOMS male = 60%.  DOMS female = 78%.  HR males = 153 (17.4).  HR females = 151 (18.3).  Exertion 85%HRmax males = 43%  Exertion 85%HRmax females = 37% exceeded the maximum.  ≥95% of participants perform the test without adverse events. | 2-km walk was considered safe. |
| Oja et al., 1991^[34]^ | 159 adults  Males (n=79)  Females (n=80)  Healthy | 41 ± 13.7  42.9 ± 14.0  39.1 ± 13.4 | 2-km walk | -RPE: 0-10 points (mean (SD))  -HR (bpm) (mean (SD))  -Exertion perceived  -Adverse events (%) | RPE males = 3.0 (1.8).  RPE females = 2.9 (1.5).  HR males = 153.1 (22.2).  HR females = 153.8 (16.0).  Exertion perceived as “moderate”.  ≥70% of participants perform the test without adverse events. | 2-km walk was considered safe. |
| Laukkanen et al., 1992^(8)^ | 277 adults  Males (n = 124)  Females (n = 153)  Healthy | 44.5 ± 10.3  44.5 ± 10.2  44.5 ± 10.5 | 2-km walk | -RPE: Borg scale 0-10 (mean (SD))  -HR (bpm) (mean (SD)) | RPE = 3.1 (2.4).  Exertion perceived as “moderate”.  HR males = 139.1 (19.1)  HR females = 143.5 (17.1). | 2-km walk was considered safe. |
| Anderson & Dal Corso, 2016^[29]^ | 20 adults  Healthy | 52  41 - 66 | 6-min walk | -HR (bpm) (mean (SD))  -Dyspnea (Borg scale 0-10) (mean (range))  -Leg fatigue (Borg scale 0-10) (mean (range))  -Adverse events (%) | HR = 128 (25.0).  Borg scale dyspnea = 2 (1-3).  Borg scale leg fatigue = 2 (1-3).  100% of participants perform the test without adverse events. | 6-min walk seems to be safe. |
| España-Romero et al., 2010^[39]^ | 58 children  80 adolescents  Healthy | 6 - 11.9  12 - 18 | 20-m shuttle run | - Adverse events (%)  -DOMS questionnaire (%) | Sick feeling =0.8%.  Injury = 0.8%.  71.2% experienced some degree of DOMS; of them, the 29.3% assumed that the 20 m shuttle run test could be the cause. | 20-m shuttle run was considered safe. |
| Lamoneda et al., 2020^[31]^ | 386 adolescents  Males (n =189)  Females (n = 197)  Healthy | 14.5 ± 1.6  14.4 ± 1.6  14.5 ± 1.6 | 20-m shuttle run music  vs  20-m shuttle run | -RPE: Borg scale 0-10 (mean (SD))  -HR (bpm) (mean (SD)) | RPE = 8.3 (2.0) vs. 7.9 (2.2).  HR = 177.1 (30.6) vs. 177.7 (29.2). | 20-m shuttle run music and 20-m shuttle run were considered safe. |
| *Musculoskeletal fitness (isometric)* |  |  |  |  |  |  |
| Hébert et al., 2011^[41]^ | 33 children  41 adolescents  Males (n = 37)  Females (n = 37)  Healthy | 4 - 9.9  10 - 17.5 | Handgrip | -Adverse events (%) | 100% of participants perform the test without adverse events (i.e., pain or discomfort). | Handgrip was considered safe. |
| España-Romero et al., 2010^[39]^ | 58 children  80 adolescents  Healthy | 6 - 11.9  12 - 18 | Handgrip | -Instrument allergy  -Adverse events (%)  -DOMS questionnaire (%) | Instrument allergy = 0%.  Injury = 0%,  sick feeling = 0%,  pain in hand or forearm = 0.8%.  DOMS= 0%. | Handgrip was considered safe. |
| Suni et al., 1998^[20]^ | 600 adults  Males (n = 246)  Females (n = 354)  Healthy | 37 - 57  47.0 ± 7.9 | Handgrip | -DOMS questionnaire (%)  -HR (bpm) (mean (SD))  - Exertion 85%HRmax (%)  -Adverse events (%) | DOMS male = 0%  DOMS female = 0%  HR males = 95 (15.2).  HR females = 92 (15.3).  Exertion 85%HRmax = 0% participants exceeded de maximum percentage.  ≥95% of participants perform the test without adverse events. | Handgrip was considered safe. |
| *Musculoskeletal fitness (explosive)* |  |  |  |  |  |  |
| Smits-Engelsman et al., 2020^[42]^ | 80 children  Males (n = 39)  Females (n = 41)  Healthy | 9.2 ± 1.1 | Standing long jump | -Adverse events (%) | 100% of participants perform the test without adverse events (injuries). | Standing long jump seems to be safe. |
| España-Romero et al., 2010^[39]^ | 58 children  80 adolescents  Healthy | 6 - 11.9  12 - 18 | Standing long jump | -Adverse events (%)  -DOMS questionnaire (%) | Sick feeling = 0.  Injury = 0%.  71.2% experienced some degree of DOMS between differences tests that could be the cause (i.e. 20-m shuttle run or standing long jump). | Standing long jump seems to be safe. |
| Suni et al., 1998^[20]^ | 600 adults  Males (n = 246)  Females (n = 354)  Healthy | 37 - 57  47.0 ± 7.9 | Vertical jump | -DOMS questionnaire (%)  -HR (bpm) (mean (SD))  -Exertion 85%HRmax (%)  -Adverse events (%) | DOMS male = 0%.  DOMS female = 0%.  HR males = 110 (16.3).  HR females = 112 (17.4).  Exertion 85%HRmax = 0% participants exceeded de maximum percentage.  ≥95% of participants perform the test without adverse events. | Vertical jump seems to be safe. |
| *Musculoskeletal fitness (endurance)* |  |  |  |  |  |  |
| Bruggeman et al., 2020^[28]^ | 40 Children  Males (n = 23)  Females (n = 17)  Healthy | 12.8 ± 1.9  13.3 ± 1.7  12.1 ± 2.0 | 45-s squat | -Rating of perceived exertion (RPE) (Borg scale 0-10) (mean (SD))  -HR (bpm) (mean (SD))  -Adverse events (%) | RPE = 3.4 (1.7).  HR not available data.  100% of participants perform the test without adverse events. | 45-s squat was considered safe. |
| Ito et al., 1996^[44]^ | 90 adults  Males (n = 37)  Females (n = 53)  Healthy | 44.3  46.8 | Trunk flexor endurance  Isometric back endurance | -Adverse events (%) | 100% of participants perform the test without adverse events (pain, problems).  100% of participants perform the test without adverse events (pain, problems). | Trunk flexor endurance and isometric back endurance tests seem to be safe. |
| Suni et al., 1998^[20]^ | 600 adults  Males (n = 246)  Females (n = 354)  Healthy | 37 - 57  47.0 ± 7.9 | Isometric back endurance  Modified push-ups | -DOMS questionnaire (%)  -HR (bpm) (mean (SD))  -Exertion 85%HRmax (%)  -Adverse events (%) | DOMS male = 0%.  DOMS female = 0%.  HR males = 112 (18.3).  HR females = 121 (19.8).  Exertion 85%HRmax = 1.28% participants exceed the maximum.  ≥90% of participants perform the test without adverse events.  DOMS male = 0%.  DOMS female = 0%.  HR males = 140 (18.0).  HR females = 144 (17.5).  Exertion 85%HRmax = 9.2% participants exceed the maximum.  ≥90% of participants perform the test without adverse events. | Isometric back endurance was considered safe.  Modified push-ups was considered safe. |
| *Motor fitness (balance)* |  |  |  |  |  |  |
| Smits-Engelsman et al., 2020^[42]^ | 80 children  Males (n = 39)  Females (n = 41)  Healthy | 9.2 ± 1.1 | Single-leg stand  Dynamic balance | -Adverse events (%) | 100% of participants perform the test without adverse events.  100% of participants perform the test without adverse events. | Single-leg stand and dynamic balance tests were considered safe. |
| Suni et al., 1998^[20]^ | 600 adults  Males (n = 246)  Females (n = 354)  Healthy | 37 - 57  47.0 ± 7.9 | Single-leg stand | -DOMS questionnaire (%)  -HR (bpm) (mean (SD))  -Exertion 85%HRmax (%)  -Adverse events (%) | DOMS male = 0%.  DOMS female = 0%.  HR males = 100 (16.1).  HR females = 94 (16.0).  Exertion 85%HRmax = 0% participants exceeded de maximum. percentage.  ≥90% of participants perform the test without adverse events. | Single-leg stand was considered safe. |

Adverse events refer to falls, sick feeling, pain and/or injuries.

**REFERENCES**

20. Suni JH, Miilunpalo, S. I., Asikainen, T. M., Laukkanen, R. T., Oja, P., Pasanen, M. E., & Vuori, I. M. Safety and feasibility of a health-related fitness test battery for adults. Phys Ther. 1998;78(2):134-48.

28. Bruggeman BS, Vincent, H. K., Chi, X., Filipp, S. L., Mercado, R., Modave, F., & Bernier, A. Simple tests of cardiorespiratory fitness in a pediatric population. Plos one. 2020;15(9).

29. José A, & Dal Corso, S. Step tests are safe for assessing functional capacity in patients hospitalized with acute lung diseases. J Cardiopulm Rehabil Prev. 2016;36(1):56-61.

30. Borel B, Fabre, C., Saison, S., Bart, F., & Grosbois, J. M. An original field evaluation test for chronic obstructive pulmonary disease population: the six-minute stepper test. Clin Rehabil. 2010;24(1):82-93.

31. Lamoneda J, Huertas-Delgado, F. J., & Cadenas-Sanchez, C. Feasibility and concurrent validity of a cardiorespiratory fitness test based on the adaptation of the original 20 m shuttle run: The 20 m shuttle run with music. J Sports Sci. 2021;39(1):57-63.

32. Amado-Pacheco JC, Prieto-Benavides DH, Correa-Bautista JE, García-Hermoso A, Agostinis-Sobrinho C, María Alonso-Martínez A., et al. Feasibility and reliability of physical fitness tests among colombian preschool children. Int J Environ Res Public Health. 2019;16(17):3069.

34. Oja P, Laukkanen, R., Pasanen, M., Tyry, T., & Vuori, I. A 2-km walking test for assessing the cardiorespiratory fitness of healthy adults. Int J Sports Med. 1991;12(4):356-62.

35. Laukkanen RM, Oja, P., Ojala, K. H., Pasanen, M. E., & Vuori, I. M. Feasibility of a 2-km walking test for fitness assessment in a population study. Scand J Med Sci Sports. 1992;20(2):119-26.

39. España-Romero V, Artero EG, Jimenez-Pavón D, Cuenca-Garcia M, Ortega FB, Castro-Piñero J, et al. Assessing health-related fitness tests in the school setting: reliability, feasibility and safety; the ALPHA Study. Int J Sports Med. 2010;31(7):490-7.

40. Aadahl M, Zacho, M., Linneberg, A., Thuesen, B. H., & Jørgensen, T. Comparison of the Danish step test and the watt-max test for estimation of maximal oxygen uptake: the Health 2008 study. Eur J Prev Cardiol. 2013;20(6):1088-94.

41. Hébert LJ, Maltais, D. B., Lepage, C., Saulnier, J., Crête, M., & Perron, M. . Isometric muscle strength in youth assessed by hand-held dynamometry: A feasibility, reliability, and validity study: A feasibility, reliability, and validity study. Pediatr Phys Ther. 2011;23(3):289-99.

42. Smits-Engelsman B, Bonney, E., Neto, J. L. C., & Jelsma, D. L. Feasibility and content validity of the PERF-FIT test battery to assess movement skills, agility and power among children in low-resource settings. BMC Public Health. 2020;20(1):1-11.

44. Ito T, Shirado, O., Suzuki, H., Takahashi, M., Kaneda, K., & Strax, T. E. Lumbar trunk muscle endurance testing: an inexpensive alternative to a machine for evaluation. Arch Phys Med Rehabil. 1996;77(1):75-9.
