# Supplementary material for: Feasibility and Safety of Field-Based Physical Fitness Tests: A Systematic Review
Source: Sports Med Open. 2025 Jan 24;11:8. doi: 10.1186/s40798-024-00799-1 (PMC11759754; doi:10.1186/s40798-024-00799-1)
Supplement: Supplementary file 8 — Supplementary Material 8. [file 40798_2024_799_MOESM8_ESM.docx]

**Supplementary Table S7.** Description for each one of the field-based physical fitness tests included in the systematic review.

| **Study** | **Fitness Component** | **Field-based fitness test** | **Equipment** |  | **Test procedure** |  | **Scoring** |
| --- | --- | --- | --- | --- | --- | --- | --- |
| Anderson & Dal Corso, 2016^[29]^  Langhammer & Stanghelle, 2018^[33]^ | Cardiorespiratory fitness | 6-min walk | - A well ventilated room, non-slip hard surface  - Tape measure  - Cones (not mandatory).  - A rectangular shape, divided into 5 yards length measuring a total of 50 yards (45.7 m). |  | The participant has to walk for 6-minutes as fast as possible at a steady pace without running. |  | Total distance (meters). |
| Oja et al., 1991^[34]^  Laukkanen et al., 1992^[35]^  Suni et al., 1998^[20]^ | Cardiorespiratory fitness | 2-km walk | - 500-m stretch of flat dirt road  - Heart rate (HR) monitor (Sport Tester PE 3000, Polar Electro Oy, Finland).  - Flat asphalt-gravel road  - The 2-km test course was marked with signs every 50-m.  - HR monitor (Sport Tester  PE 3000, Polar Electro Oy, Finland).  - 500-m stretch of flat dirt road  - HR monitor (Polar Sport Tester, Polar Electro A, Professorintie 5. 90440 kernpele. Finland). |  | The participant has to walk for 2-km as fast as possible at a steady pace without running. |  | Age, body mass index (BMI), total time (minutes) and mean HR during the last 30-seconds of the walk. Vo2max (ml kg−1 min−1) by Oja’s equation**^1^.**  Age, body mass index (BMI), total time (minutes) and HR at the end.  Vo2max (ml kg−1 min−1) by Oja’s equation**^1^**.  Age, body mass index (BMI), total time (minutes) and mean HR during the last 30-seconds of the walk. Vo2max (ml kg−1 min−1) by Oja’s equation**^1^.** |
| Fjortoft et al., 2011^[36]^ | Cardiorespiratory fitness | 6-min run | - A gymnasium or a space large enough to mark a rectangle of 9 x 18-m  - Masking tape  - Tape measure  - Stopwatch. |  | The participant has to run or walk around a marked area for 6-minutes. |  | Total distance (meters). |
| España-Romero et al., 2010^[39]^  Lamoneda et al., 2020^[31]^  Cadenas-Sanchez et al., 2014^[37]^  Cadenas-Sánchez et al., 2016^[38]^  Amado-Pacheco et al., 2019^[32]^ | Cardiorespiratory fitness | 20-m shuttle run | - A gymnasium or a space large enough to mark a distance of 20-m  - 4 cones  - Tape measure  - Audio track with the test protocol and an audio player. |  | The participant has to run from one line to another at a distance of 20-m, changing direction at the rhythm indicated by a sound signal that will gradually accelerate. One attempt is made.  For children and adolescents and adults, the speed is imposed by an acoustic signal starting at 8.5 km/h and increasing every minute by 0.5 km/h.  For preschoolers, the initial speed is modified and starts at 6.5 km/h, and will be increased by 0.5 km/h/min (1-min equals 1 bearing/staging). |  | Age and number of stages completed (for children, adolescents and adults).  Vo2max (ml kg−1 min−1) by Leger’s equation**^2^.**  Age and number of laps as well as stages completed (for preschooler).  Vo2max (ml kg−1 min−1) by 20-m shuttle run test PREFIT equation**^3^.** |
| Lamoneda et al., 2020^[31]^ | Cardiorespiratory fitness | 20-m shuttle run music | - A gymnasium or a space large enough to mark a distance of 20 m  - 4 cones  - Tape measure  - Audio track with the test protocol and an audio player. |  | The only difference is the incorporation of audio tracks (i.e., music) into the original file. A different song is played every single minute. One attempt is made. |  | Age and number of stages completed.  Vo2max (ml kg−1 min−1) by Leger’s equation**^2^.** |
| Langhammer & Stanghelle, 2018^[33]^ | Cardiorespiratory fitness | 2-min step | - A non-slip surface  - Masking tape  - Tape measure  - Stopwatch. |  | The participant has to stand next to a wall and lift each knee up to overlap a previously defined mark on the wall as many times as possible for 2-minutes. One attempt is made. |  | Number of times right knee reaches the marked height in 2-minutes. |
| Bruggeman et al., 2020^[28]^ | Cardiorespiratory fitness | 3-min step | - A non-slip surface  - A metronome (*no brand specified*)  - 12-inch (30-cm) step bench. |  | The participant has to step up and down on the step bench at the given rate for a total of 3-minutes. One attempt is made. |  | Scoring = HR from T0 to T4  Vo2max (ml kg−1 min−1) by Jacks equation**^4^.** |
| Borel et al., 2010^[30]^ | Cardiorespiratory fitness | 6-min step | - A non-slip surface  - Stepper motor (V Fit S 726, Vivace Fitness, Durham, United Kingdom).  - A step bench (20-cm). |  | The participant has to step up and down on the stepper at the given rate for a total of 6-minutes. One attempt is made. |  | Number of steps in 6-minutes. |
| Aadahl et al., 2012^[40]^ | Cardiorespiratory fitness | Danish step | - A non-slip surface  - Software for guiding the cadence of the step and calculating results  - Four different step bench heights were used: 20-cm, 25-cm, 30-cm and 35-cm. |  | The participant has to step up and down on the step bench keeping the pace for as long as possible at the given rate. One attempt is made. |  | Total time spent (minutes). |
| Anderson & Dal Corso, 2016^[29]^ | Cardiorespiratory fitness | Chester step | - A non-slip surface  - Cadence tape  - 20-cm step bench  - Pulse oximeter (9500, Nonin, Plymouth, MN). |  | The participant has to step up and down on the bench at the given rate for 5 stages of 2-minutes (10-minutes). One attempt is made. |  | Total number of steps completed. |
| Anderson & Dal Corso, 2016^[29]^ | Cardiorespiratory fitness | Modified incremental step | - A non-slip surface  - Cadence tape  - 20-cm step bench  - Pulse oximeter (9500, Nonin, Plymouth, MN). |  | The participant has to step up and down on the bench keeping the pace with 10 steps per minute and with constant increments of 1 step every 30-seconds. One attempt is made. |  | Total number of steps completed. |
| España-Romero et al., 2010^[39]^  Hébert et al., 2011^[41]^  Cadenas-Sánchez et al., 2016^[38]^  Amado-Pacheco et al., 2019^[32]^  Suni et al., 1998^[20]^ | Musculoskeletal fitness  *(Maximal*  *isometric strength)* | Handgrip | - Digital dynamometer with adjustable grip (TKK 5101 Grip D; Takey, Tokio Japan)  - Analogue dynamometer with adjustable grip (TKK 5001 Grip A, analogue model, measuring range 0-100; Takey, Tokio Japan).  *Model of dynamometer used is not specified*. |  | The participant has to squeeze the dynamometer slowly and continuously for at least 2-seconds, performing the test twice (alternately with both hands) with the optimal grip setting and allowing a short rest between measurements. The test is performed twice per hand. |  | The maximum score for each hand (kilograms). |
| España-Romero et al., 2010^[39]^  Fjortoft et al., 2011^[36]^  Smits-Engelsman et al., 2020^[42]^  Cadenas-Sánchez et al., 2016^[38]^  Amado-Pacheco et al., 2019^[32]^ | Musculoskeletal fitness  *(Explosive strength)* | Standing long jump | - Non-slip hard surface  - Stick or flat spike  - Tape measure  - Tape  - Cones (not mandatory). |  | The participant has to jump as far as possible with feet together (separate from each other approximately at the shoulder’s width), and he/she bends his/her knees with arms in front of the body and parallel to the ground. swings arms are allowed. For children and adolescents and adults two attempts are performed.  For preschoolers three attempts are performed. |  | The distance between the starting line and the landing position. The best attempt is recorded (centimeters). |
| Suni et al., 1998^[20]^ | Musculoskeletal fitness  *(Explosive strength)* | Vertical jump | - Jump-and-reach board  - Tape measure. |  | The participant has to jump vertically as high as possible using arms and legs projecting the body upwards. Two attempts are made. |  | The difference between the reach height and the jump height (centimeters). The best attempt is recorded. |
| Fjortoft et al., 2011^[36]^ | Musculoskeletal fitness  *(Explosive strength)* | Medicine ball push | - Medicine ball (1kg). |  | The participant has to push a medicine ball (1 kg) with two hands as far as possible. Two attempts are made. |  | The distance achieved (meters). The best attempt is recorded. |
| Boyer et al., 2013^[43]^ | Musculoskeletal fitness  *(Strength-endurance)* | Partial curl-ups, 60-s, 90-s and unlimited plank | - A non-slip surface  - A mat  - Audio track with the test protocol and an audio player. |  | For the Fitnessgram partial curl-up, participant has to slide their hands forward maintaining a cadence of 20 partial curl-ups per minute. One attempt is made.  For the CHMS partial curl-ups, participant has to slide their hands forward and perform as many partial curl-ups as possible in one minute. One attempt is made.  For the 60-seconds, 90-seconds and unlimited plank, participant has to maintain a static prone position with only forearms and toes touching the ground. One attempt is made. |  | Number of partial curl-ups completed (maximum 75 repetitions).  Number of partial curl-ups completed in 1 minute.  Different maximum time limits were evaluated and recorded across three distinct cycles of testing (60-seconds, 90-seconds and unlimited plank). |
| Ito et al., 1996^[44]^ | Musculoskeletal fitness  *(Strength-endurance)* | Trunk flexor endurance | - Non-slip hard surface and  - Stopwatch. |  | The participant has to lie in a supine position and to raise the lower extremities with 90° flexion of the hip and knee joints and maintaining the positions for as long as possible. One attempt is made. |  | The performance time (seconds). |
| Ito et al., 1996^[44]^  Suni et al., 1998^[20]^ | Musculoskeletal fitness  *(Strength-endurance)* | Isometric back endurance | - Non-slip hard surface  - Small pillow  - Stopwatch.  - Non-slip hard surface  - Stopwatch. |  | The participant has to lie in a prone position while holding the sternum off the floor and maintain their maximum flexion of cervical spine, with pelvic stabilization through gluteal muscle contraction for as long as possible. One attempt is made. |  | The performance time (seconds).  The performance time (seconds) of the task up to 4-minutes. |
| Suni et al., 1998^[20]^ | Musculoskeletal fitness  *(Strength-endurance)* | Modified  push-ups | - Non-slip hard surface and  - Stopwatch. |  | The participant has to place his/her hands on the floor slightly wider than shoulder width apart and knees apart, then bend his/her elbows and lower their chest until their chin reaches the floor, then slowly return to the starting position. One attempt is made. |  | Number of push-ups completed in 40-seconds. |
| Langhammer & Stanghelle, 2018^[33]^ | Musculoskeletal fitness  *(Strength-endurance)* | Arm curl | - Non-slip hard surface  - Chair without armrests  - Stopwatch  - Weights: 5lb (2.27 kg) for women and 8lb (3.63 kg) for men. |  | The participant has to sit in the chair and hold the weight in the hand using a suitcase grip. The arm will curve upwards through the full range of motion and descends returning to the starting position. One attempt is made. |  | Number of biceps curls completed in 30-seconds. |
| Langhammer & Stanghelle, 2018^[33]^ | Musculoskeletal fitness  *(Strength-endurance)* | 30-s  sit-to-stand | - Non-slip hard surface  - Chair with a seat height of 45-cm without armrests  - Stopwatch. |  | The participant has to stand up all the way until his/her knee and hip are fully extended and sit down as many times as possible for 30-seconds. One attempt is made. |  | Number of stands performed in 30-seconds. |
| McAllister & Palombaro, 2019^[27]^ | Musculoskeletal fitness  *(Strength-endurance)* | Modified 30-s sit-to-stand | - Non-slip hard surface  - Chair with a seat height of 45-cm and standard armrests were designated to be used in the study and  - Stopwatch. |  | The participant has to seat in the middle of the chair, with his/her back straight, and feet shoulder width apart and place his/her hands on the armrest. At the "go" signal, the participant has to stand up and sit down as many times as possible for 30-seconds. One attempt is made. |  | Number of stands performed in 30-seconds. |
| Bruggeman et al., 2020^[28]^ | Musculoskeletal fitness  *(Strength-endurance)* | 45-s squat | - Non-slip hard surface  - A metronome (*no brand specified*) and counted out loud by the coordinator. |  | The participant has to complete 30-squats in 45-seconds, paced by a metronome and counted out loud by the coordinator. One attempt is made. |  | Scoring = HR from P0 to P2 by the Ruffier**^5^** and Dickson**^6^** indices. |
| Amado-Pacheco et al., 2019^[32]^ | Musculoskeletal fitness  *(Flexibility)* | Sit and reach | - Testing box**^7^**  - Two gymnasium benches  - A piece of rubber matting about four feet square. |  | The participant has to sit on the floor with legs extended and feet resting on the base of the box, tries to reach as far forward as possible with his/her hands together on the scale located on the top surface of the test box. Two attempts are made. |  | The score is the most distant line touched by the fingertips of both hands over the scale (centimeters).  The best attempt is recorded. |
| Langhammer & Stanghelle, 2018^[33]^ | Musculoskeletal fitness  *(Flexibility)* | Chair sit and reach | - Clean, non-slip surface  - Chair with a seat height of 45-cm without armrests  - Stopwatch  - 4 cones  - 45-cm ruler. |  | The participant has to sit on the edge of a chair, stretches the leg while maintaining a 90° ankle flexion and tries to reach the toes with the hands.  Two attempts with each leg are made. |  | The score is taken in centimeters.  The best attempt of each leg is recorded. |
| Langhammer & Stanghelle, 2018^[33]^ | Musculoskeletal fitness  *(Flexibility)* | Back scratch | - Tape measure. |  | The participant has to bring one hand over the same shoulder and the other hand goes to touch the middle of the back, trying to make both hands touch each other. Two attempts with each arm are made. |  | The score is the distance between the extended middle fingers (centimeters).  The best attempt for each arm is recorded. |
| Fjortoft et al., 2011^[36]^ | Motor fitness  *(Speed)* | 20-m run | - Masking tape  - Tape measure  - Stopwatch. |  | The participant has to run 20-m as fast as possible. Two attempts are performed. |  | The performance time (seconds).  The best attempt is recorded. |
| Cadenas-Sánchez et al., 2016^[38]^  Amado-Pacheco et al., 2019^[32]^ | Motor fitness  *(Agility)* | 4 x 10-m shuttle run | - Clean, non-slip surface  - Masking tape  - Stopwatch  - 4 cones  - Tape measure. |  | The participant has to run and turn at maximum speed and complete the distance (4x10-m). Two attempts are performed |  | The performance time (seconds).  The best attempt is recorded. |
| Fjortoft et al., 2011^[36]^ | Motor fitness  *(Agility)* | 10 × 5-m shuttle run | - Clean, non-slip surface  - Masking tape  - 4 cones  - Tape measure  - Stopwatch. |  | The participant has to run and turn at maximum speed and complete the distance (10x5-m). One attempt is performed. |  | The performance time (seconds). |
| Langhammer & Stanghelle, 2018^[33]^ | Motor fitness  *(Agility)* | 2.45-m time up & go | - Clean, non-slip surface  - Chair with a seat height of 45-cm without armrests  - Stopwatch  - Cones  - Tape measure. |  | The participant has to start from a sitting position. The time it takes to stand up, walk to a cone 2.45-m away, turn around and sit down again is measured. Two attempts are performed. |  | The performance time (seconds). The best attempt is recorded. |
| Cadenas-Sánchez et al., 2016^[38]^  Smits-Engelsman et al., 2020^[42]^  Suni et al., 1998^[20]^ | Motor fitness  *(Balance)* | Single-leg stand | - Non-slip hard surface and  - Stopwatch. |  | In preschoolers, the participant has to stand on one leg on the floor, with eyes open and the free leg flexed at the knee forwards, as long as possible. One attempt is performed with each leg.  In children, the participant has to stand on one leg on the floor, with eyes open and the free leg flexed at the knee to the back, grasping the foot against the buttocks, as long as possible. Two attempts are made with each leg.  In adults, the participant has to stand on one leg on the floor, with eyes open and the free leg rests with the heel on the inside of the knee joint of the supporting leg and turning the thigh outwards, as long as possible. Two attempts are made with each leg. |  | The mean time of right and left leg combined (seconds).  Maximum time was 30-seconds for each leg (no rest between legs needed).  The mean time of right and left leg combined (seconds).  Duration of task up to 60-seconds. |
| Smits-Engelsman et al., 2020^[42]^ | Motor fitness  *(Balance)* | Dynamic balance | - Non-slip hard surface  - Agility ladder  - Stopwatch. |  | The participant has to grasp his/her unsupported foot while performing 5 small jumps with each leg in the agility ladder (dynamic). Two attempts are made with each leg. |  | Number of jumps performed correctly (10 points is the maximum, 5 points per leg). |
| Fjortoft et al., 2011^[36]^ | Motor fitness  *(Multi-skills)* | Jumping a distance of 7-m on 1 foot  Jumping a distance of 7-m on 2 feet | - Non-slip hard surface  - Masking tape  - Ruler and  - Stopwatch. |  | The participant has to jump a distance of 7-m on one foot (he/she is free to choose which foot) as fast as possible. Two attempts are made  The participant has to jump a distance of 7-m on two feet together as fast as possible. Two attempts are made. |  | Total time to perform the distance (seconds).  The best attempt is recorded.  Total time to perform the distance (seconds).  The best attempt is recorded. |

**^1^Oja equation** = for male: 184.9 - 4.65*(time) - 0.22*(HR) - 0.26*(age) -105*(BMI), for female: l16.2 - 2.98*(time) - 0.l l*(HR) - 0.14*(age) - 0.39*(BMI); **^2^Leger equation** = for 6 to 18 years: 31.025 + 3.238*[speed(km*h^-1^)] - 3.248*[age(years)] + 0.1536*[speed]*[years], for 18 years and older: 24.4 + 6.0*[speed(km*h^-1^)]; **^3^20mSRT-PREFIT equation** = 44.657 + 1.795* [speed(km*h^-1^)] − 2.601*[age(years)] + 0.0852* [speed]*[years]; **^4^Jacks equation** = 2.045 + (height in inch*0.062) +100*[1/3*(T1+T2+T3) / T0]*(-0.411) + (T0*0.011), where the scoring: T0 = resting HR before 3-min step test, T3 = HR immediately upon completion, T4 = HR after one minute of rest; **^5^Ruffier index** = (P0 + P1 + P2–200) / 10; **^6^Dickson index** = ((P1–70) + 2*(P2-P0))/10, where the scoring: P0 = heart rate (HR) at rest, P1 = HR immediately upon completion, P2 = HR after one minute of rest; **^7^Testing box measures** = The box had a projecting measuring scale that extended 22-cm towards the subject. This device permitted a scoring range from 0-cm, suggesting very low flexibility, to 50-cm, suggesting very high flexibility.

BMI = Body Max Index; CHMS = Canadian Health Measures Survey; RPE = Rating of Perceived Exertion; Vo2max. = Maximum Volume of Oxygen Uptake;

**REFERENCES**

20. Suni JH, Miilunpalo, S. I., Asikainen, T. M., Laukkanen, R. T., Oja, P., Pasanen, M. E., & Vuori, I. M. Safety and feasibility of a health-related fitness test battery for adults. Phys Ther. 1998;78(2):134-48.

27. McAllister LS, & Palombaro, K. M. Modified 30-second sit-to-stand test: reliability and validity in older adults unable to complete traditional sit-to-stand testing. J Geriatr Phys Ther. 2020;43(3):153-8.

28. Bruggeman BS, Vincent, H. K., Chi, X., Filipp, S. L., Mercado, R., Modave, F., & Bernier, A. Simple tests of cardiorespiratory fitness in a pediatric population. Plos one. 2020;15(9).

29. José A, & Dal Corso, S. Step tests are safe for assessing functional capacity in patients hospitalized with acute lung diseases. J Cardiopulm Rehabil Prev. 2016;36(1):56-61.

30. Borel B, Fabre, C., Saison, S., Bart, F., & Grosbois, J. M. An original field evaluation test for chronic obstructive pulmonary disease population: the six-minute stepper test. Clin Rehabil. 2010;24(1):82-93.

31. Lamoneda J, Huertas-Delgado, F. J., & Cadenas-Sanchez, C. Feasibility and concurrent validity of a cardiorespiratory fitness test based on the adaptation of the original 20 m shuttle run: The 20 m shuttle run with music. J Sports Sci. 2021;39(1):57-63.

32. Amado-Pacheco JC, Prieto-Benavides DH, Correa-Bautista JE, García-Hermoso A, Agostinis-Sobrinho C, María Alonso-Martínez A., et al. Feasibility and reliability of physical fitness tests among colombian preschool children. Int J Environ Res Public Health. 2019;16(17):3069.

33. Langhammer B, & Stanghelle, J. K. Senior fitness test; a useful tool to measure physical fitness in persons with acquired brain injury. Brain Inj. 2019;33(2):183-8.

34. Oja P, Laukkanen, R., Pasanen, M., Tyry, T., & Vuori, I. A 2-km walking test for assessing the cardiorespiratory fitness of healthy adults. Int J Sports Med. 1991;12(4):356-62.

35. Laukkanen RM, Oja, P., Ojala, K. H., Pasanen, M. E., & Vuori, I. M. Feasibility of a 2-km walking test for fitness assessment in a population study. Scand J Med Sci Sports. 1992;20(2):119-26.

36. Fjørtoft I, Pedersen, A. V., Sigmundsson, H., & Vereijken, B. Measuring physical fitness in children who are 5 to 12 years old with a test battery that is functional and easy to administer. Phys Ther. 2011;91(7):1087-95.

37. Cadenas-Sanchez C, Alcántara-Moral, F., Sanchez-Delgado, G., Mora-Gonzalez, J., Martinez-Tellez, B., Herrador-Colmenero, M., & Ortega, F. B. Assessment of cardiorespiratory fitness in preschool children: adaptation of the 20 metres shuttle run test. Nutr Hosp. 2014;30(6):1333-43.

38. Cadenas-Sanchez C, Martinez-Tellez B, Sanchez-Delgado G, Mora-Gonzalez J, Castro-Piñero J, Löf M, et al. Assessing physical fitness in preschool children: Feasibility, reliability and practical recommendations for the PREFIT battery. J Sci Med Sport. 2016;19(11):910-5.

39. España-Romero V, Artero EG, Jimenez-Pavón D, Cuenca-Garcia M, Ortega FB, Castro-Piñero J, et al. Assessing health-related fitness tests in the school setting: reliability, feasibility and safety; the ALPHA Study. Int J Sports Med. 2010;31(7):490-7.

40. Aadahl M, Zacho, M., Linneberg, A., Thuesen, B. H., & Jørgensen, T. Comparison of the Danish step test and the watt-max test for estimation of maximal oxygen uptake: the Health 2008 study. Eur J Prev Cardiol. 2013;20(6):1088-94.

41. Hébert LJ, Maltais, D. B., Lepage, C., Saulnier, J., Crête, M., & Perron, M. . Isometric muscle strength in youth assessed by hand-held dynamometry: A feasibility, reliability, and validity study: A feasibility, reliability, and validity study. Pediatr Phys Ther. 2011;23(3):289-99.

42. Smits-Engelsman B, Bonney, E., Neto, J. L. C., & Jelsma, D. L. Feasibility and content validity of the PERF-FIT test battery to assess movement skills, agility and power among children in low-resource settings. BMC Public Health. 2020;20(1):1-11

43. Boyer C, Tremblay, M., Saunders, T., McFarlane, A., Borghese, M., Lloyd, M., & Longmuir, P. Feasibility, validity, and reliability of the plank isometric hold as a field-based assessment of torso muscular endurance for children 8–12 years of age. Pediatr Exerc Sci. 2013;25(3):407-22.

44. Ito T, Shirado, O., Suzuki, H., Takahashi, M., Kaneda, K., & Strax, T. E. Lumbar trunk muscle endurance testing: an inexpensive alternative to a machine for evaluation. Arch Phys Med Rehabil. 1996;77(1):75-9.
