# Supplementary material for: Feasibility and Safety of Field-Based Physical Fitness Tests: A Systematic Review
Source: Sports Med Open. 2025 Jan 24;11:8. doi: 10.1186/s40798-024-00799-1 (PMC11759754; doi:10.1186/s40798-024-00799-1)
Supplement: Supplementary file 9 — Supplementary Material 9. [file 40798_2024_799_MOESM9_ESM.docx]

**Supplementary Table S8.** Items to establish feasibility reported by evaluators and participants^$^.

| **Study** | **Participants performing the test (%)** | **Evaluators (n)** | **Participants measured (n)** | **Tests** | **Time to prepare test** | **Time to**  **perform test** | **Easy to administer** |
| --- | --- | --- | --- | --- | --- | --- | --- |
| McAllister & Palombaro, 2019^[27]^ | 82% |  |  | Modified 30-s sit-to-stand |  | 30 seconds |  |
| Bruggeman et al., 2020^[28]^ |  |  |  | 45-s squat and 3-min step |  | 45 seconds /  3 minutes | Yes |
| Anderson & Dal Corso, 2016^[29]^ |  |  |  | Chester step, modified incremental step and 6-min walk |  | 10 / 12 / 6 minutes |  |
| Borel et al., 2010^[30]^ | 100% |  |  | 6-min step |  | 6 minutes |  |
| Lamoneda et al., 2020^[31]^ |  |  |  | 20-m shuttle run music vs. 20-m shuttle run |  |  |  |
| Aadahl et al., 2012^[40]^ | 98.8% |  |  | Danish step |  | 6 minutes |  |
| Langhammer & Stanghelle, 2018^[33]^ | 100% |  |  | 2-min step, 6-min walk and 30-s sit-to-stand |  | 2 / 2 minutes / 30 seconds |  |
| Oja et al., 1991^[34]^ | 100% |  |  | 2-km walk |  | 15.2 /16.9 minutes (mean for males /females) |  |
| Laukkanen et al., 1992^[35]^ | 96.4% | 2 |  | 2-km walk |  | 15 to 18 minutes |  |
| Suni et al., 1998^[20]^ | 95% | 3 |  | 2-km walk, handgrip, vertical jump, single-leg stand, isometric back endurance and modified push-ups |  | 1hr 10minutes for HRFI test-battery |  |
| Amado-Pacheco et al., 2019^[32]^ | 96% |  |  | 20-m shuttle run |  | 6.7/ 5.3 minutes (mean for males/females) |  |
| Cadenas-Sánchez et al., 2014^[37]^ |  | 2 | Groups of 4-8 (4-5 year -old) 4-5 (3-year-old) | 20-m shuttle run | 3 minutes | 8 a 10 minutes |  |
| Cadenas-Sánchez et al., 2016^[38]^ |  | 2-5 | Groups of 4-8 (same age) | 20-m shuttle run; handgrip, standing long jump, 4×10-m shuttle run and single-leg stand |  | 2.5hrs (20 participants /2 evaluators*) for PREFIT battery | Yes all, except standing long jump |
| España-Romero et al., 2010^[39]^ |  | 1 | Group of 20 | 20-m shuttle run, handgrip and standing long jump | 11minutes | 2.5hrs for Evidence-based ALPHA-Fitness test battery | Yes |
| Fjortoft et al., 2011^[36]^ | 100% |  |  | 6-min run, jumping a distance of 7-m on 1 foot/ 2 feet, medicine ball push, 20-m run and 10×5-m shuttle run |  |  | Yes |
| Hébert et al., 2011^[40]^ | 100% |  |  | Handgrip |  |  | Yes |
| Smits-Engelsman et al., 2020^[42]^ | 100% |  |  | Standing long jump, singe-leg stand and dynamic balance |  | 20-40 minutes per participants /30-50 minutes per 2 participants for PERF-FIT test battery | Yes |
| Boyer et al., 2013^[43]^ | 88-99% |  |  | Partial curl-ups, 60-s, 90-s and unlimited plank test |  | Fitnessgram /CHMS partial curl-ups (29/36 s); 60-s /90-s /unlimited time planks (41/52/56s) |  |
| Ito et al., 1996^[44]^ | 100% |  |  | Trunk flexor endurance and isometric back endurance |  | 95-89% within the time (≤5minutes) | Yes |
| **Total studies by item** | 13 | 5 | 3 |  | 2 | 16 | 7 |

**Supplementary Table S8 (continued).** Items to establish feasibility reported by evaluators and participants^$^.

| **Study** | **Understand test instructions (% yes)** | **Appropriate sport clothes (% yes)** | **Appropriate facilities**  **(% yes)** | **Reject (% yes)** | **Self-perception effort^$^ (%)** | **Self-perception fatigue^$^ (%)** | **Self-perception preference test^$^ (%)** | **Self-perception enjoyment^$^ (%)** | **Total items by study** |
| --- | --- | --- | --- | --- | --- | --- | --- | --- | --- |
| McAllister & Palombaro, 2019^[27]^ |  |  |  |  |  |  |  |  | 2 |
| Bruggeman et al., 2020^[28]^ |  |  |  |  | 100% low |  |  |  | 3 |
| Anderson & Dal Corso, 2016^[29]^ |  |  |  |  |  |  |  |  | 1 |
| Borel et al., 2010^[30]^ |  |  |  |  |  |  |  |  | 2 |
| Lamoneda et al., 2020^[31]^ |  |  |  |  | 72.4%max. for both | 56.4% less fatigue with music | 89.6% vs. 10.4% | 94% vs. 6% | 4 |
| Aadahl et al., 2012^[40]^ |  |  |  |  |  |  |  |  | 2 |
| Langhammer & Stanghelle, 2018^[33]^ |  |  |  |  |  |  |  |  | 2 |
| Oja et al., 1991^[34]^ |  |  |  |  |  |  |  |  | 3 |
| Laukkanen et al., 1992^[35]^ |  |  |  | 21% | 81% easy |  |  |  | 5 |
| Suni et al., 1998^[20]^ |  |  |  |  |  |  |  |  | 3 |
| Amado-Pacheco et al., 2019^[32]^ |  |  |  |  |  |  |  |  | 2 |
| Cadenas-Sánchez et al., 2014^[37]^ |  |  |  |  |  |  |  |  | 4 |
| Cadenas-Sánchez et al., 2016^[38]^ |  |  |  |  |  |  |  |  | 4 |
| España-Romero et al., 2010^[39]^ | ≥ 95%^#^ | ≥ 95%^#^ | ≥ 95%^#^ | 2.3%^#^ |  |  |  |  | 9 |
| Fjortoft et al., 2011^[36]^ | 100% |  |  |  |  |  |  |  | 3 |
| Hébert et al., 2011^[40]^ | 100% |  |  |  |  |  |  |  | 3 |
| Smits-Engelsman et al., 2020^[42]^ | 100% | 76% | 100% |  |  |  |  |  | 6 |
| Boyer et al., 2013^[43]^ |  |  |  |  |  |  |  |  | 2 |
| Ito et al., 1996^[44]^ |  |  |  |  |  |  |  |  | 3 |
| **Total studies by item** | 4 | 2 | 2 | 2 | 3 | 1 | 1 | 1 |  |

^$^Questions answered by participants. ^#^An acceptable level of feasibility was considered when the items were ‘positively’ answered in at least 95 % of the cases.*Estimated time to assess the whole battery with 2 evaluators, with 5 evaluators the estimated assessment time would be 1.7hrs.

**REFERENCES:**

20. Suni JH, Miilunpalo, S. I., Asikainen, T. M., Laukkanen, R. T., Oja, P., Pasanen, M. E., & Vuori, I. M. Safety and feasibility of a health-related fitness test battery for adults. Phys Ther. 1998;78(2):134-48.

27. McAllister LS, & Palombaro, K. M. Modified 30-second sit-to-stand test: reliability and validity in older adults unable to complete traditional sit-to-stand testing. J Geriatr Phys Ther. 2020;43(3):153-8.

28. Bruggeman BS, Vincent, H. K., Chi, X., Filipp, S. L., Mercado, R., Modave, F., & Bernier, A. Simple tests of cardiorespiratory fitness in a pediatric population. Plos one. 2020;15(9).

29. José A, & Dal Corso, S. Step tests are safe for assessing functional capacity in patients hospitalized with acute lung diseases. J Cardiopulm Rehabil Prev. 2016;36(1):56-61.

30. Borel B, Fabre, C., Saison, S., Bart, F., & Grosbois, J. M. An original field evaluation test for chronic obstructive pulmonary disease population: the six-minute stepper test. Clin Rehabil. 2010;24(1):82-93.

31. Lamoneda J, Huertas-Delgado, F. J., & Cadenas-Sanchez, C. Feasibility and concurrent validity of a cardiorespiratory fitness test based on the adaptation of the original 20 m shuttle run: The 20 m shuttle run with music. J Sports Sci. 2021;39(1):57-63.

32. Amado-Pacheco JC, Prieto-Benavides DH, Correa-Bautista JE, García-Hermoso A, Agostinis-Sobrinho C, María Alonso-Martínez A., et al. Feasibility and reliability of physical fitness tests among colombian preschool children. Int J Environ Res Public Health. 2019;16(17):3069.

33. Langhammer B, & Stanghelle, J. K. Senior fitness test; a useful tool to measure physical fitness in persons with acquired brain injury. Brain Inj. 2019;33(2):183-8.

34. Oja P, Laukkanen, R., Pasanen, M., Tyry, T., & Vuori, I. A 2-km walking test for assessing the cardiorespiratory fitness of healthy adults. Int J Sports Med. 1991;12(4):356-62.

35. Laukkanen RM, Oja, P., Ojala, K. H., Pasanen, M. E., & Vuori, I. M. Feasibility of a 2-km walking test for fitness assessment in a population study. Scand J Med Sci Sports. 1992;20(2):119-26.

36. Fjørtoft I, Pedersen, A. V., Sigmundsson, H., & Vereijken, B. Measuring physical fitness in children who are 5 to 12 years old with a test battery that is functional and easy to administer. Phys Ther. 2011;91(7):1087-95.

37. Cadenas-Sanchez C, Alcántara-Moral, F., Sanchez-Delgado, G., Mora-Gonzalez, J., Martinez-Tellez, B., Herrador-Colmenero, M., & Ortega, F. B. Assessment of cardiorespiratory fitness in preschool children: adaptation of the 20 metres shuttle run test. Nutr Hosp. 2014;30(6):1333-43.

38. Cadenas-Sanchez C, Martinez-Tellez B, Sanchez-Delgado G, Mora-Gonzalez J, Castro-Piñero J, Löf M, et al. Assessing physical fitness in preschool children: Feasibility, reliability and practical recommendations for the PREFIT battery. J Sci Med Sport. 2016;19(11):910-5.

39. España-Romero V, Artero EG, Jimenez-Pavón D, Cuenca-Garcia M, Ortega FB, Castro-Piñero J, et al. Assessing health-related fitness tests in the school setting: reliability, feasibility and safety; the ALPHA Study. Int J Sports Med. 2010;31(7):490-7.

40. Aadahl M, Zacho, M., Linneberg, A., Thuesen, B. H., & Jørgensen, T. Comparison of the Danish step test and the watt-max test for estimation of maximal oxygen uptake: the Health 2008 study. Eur J Prev Cardiol. 2013;20(6):1088-94.

41. Hébert LJ, Maltais, D. B., Lepage, C., Saulnier, J., Crête, M., & Perron, M. . Isometric muscle strength in youth assessed by hand-held dynamometry: A feasibility, reliability, and validity study: A feasibility, reliability, and validity study. Pediatr Phys Ther. 2011;23(3):289-99.

42. Smits-Engelsman B, Bonney, E., Neto, J. L. C., & Jelsma, D. L. Feasibility and content validity of the PERF-FIT test battery to assess movement skills, agility and power among children in low-resource settings. BMC Public Health. 2020;20(1):1-11

43. Boyer C, Tremblay, M., Saunders, T., McFarlane, A., Borghese, M., Lloyd, M., & Longmuir, P. Feasibility, validity, and reliability of the plank isometric hold as a field-based assessment of torso muscular endurance for children 8–12 years of age. Pediatr Exerc Sci. 2013;25(3):407-22.

44. Ito T, Shirado, O., Suzuki, H., Takahashi, M., Kaneda, K., & Strax, T. E. Lumbar trunk muscle endurance testing: an inexpensive alternative to a machine for evaluation. Arch Phys Med Rehabil. 1996;77(1):75-9.
