# Supplementary material for: Feasibility and Safety of Field-Based Physical Fitness Tests: A Systematic Review
Source: Sports Med Open. 2025 Jan 24;11:8. doi: 10.1186/s40798-024-00799-1 (PMC11759754; doi:10.1186/s40798-024-00799-1)
Supplement: Supplementary file 10 — Supplementary Material 10. [file 40798_2024_799_MOESM10_ESM.docx]

**Supplementary Table S9.** Items to establish safety reported by evaluators and participants^$^.

| **Study** | **Test** | **Self-perception dyspnea**^$^ | **Self-perception leg fatigue**^$^ | **Self-perception general exertion**^$^ | **DOMS**^$$^ | **Exertion 85% HRmax*** | **HR*** | **Adverse events**** | **Instrument allergy** | **Total items by study** |
| --- | --- | --- | --- | --- | --- | --- | --- | --- | --- | --- |
| Borel et al., 2010^[30]^ | 6-min step | 2.5 | 3.4 |  |  |  | **118** |  |  | 3 |
| Amado-Pacheco et al., 2019^[32]^ | 20-m shuttle run |  |  |  |  |  |  | 0 % |  | 1 |
| Bruggeman et al., 2020^[28]^ | 45-s squat/ 3-min step |  |  | 3.4/2.7 |  |  | N/A | 0% |  | 3 |
| Anderson & Dal Corso, 2016^[29]^ | Chester step/modified incremental step/ 6-min walk | 2/2/2 | 2/2/2 |  |  |  | 143/142/128 | 0.12% |  | 4 |
| Aadahl et al., 2012^[40]^ | Danish step |  |  |  |  |  |  | 0% |  | 1 |
| Suni et al., 1998^[20]^ | 2-km walk/ handgrip/vertical jump/ single-leg stand/ isometric back endurance/ modified push-ups |  |  |  | ♂60/0/0/  0/0/0  ♀78/0/0/  0/0/0 | ♂43/0/0/  0/1.3/9.2  ♀37/0/0/  0/1.3/9.2 | ♂153/95/110/  00/112/140  ♀151/92/112/  /94/121/144 | <5 % |  | 4 |
| Oja et al., 1991^[34]^ | 2-km walk |  |  | ♂ 3  ♀ 2.9 |  |  | ♂ 153  ♀ 154 | <30% |  | 3 |
| Laukkanen et al., 1992^[35]^ | 2-km walk |  |  | ♂ 3.1  ♀ 3.1 |  |  | ♂ 139  ♀ 144 |  |  | 2 |
| España-Romero et al., 2010^[39]^ | 20-m shuttle run/ handgrip /standing long jump |  |  |  | 71 |  |  | <1%**^#^** | ≥99%**^#^** | 3 |
| Lamoneda et al., 2020^[31]^ | 20-m shuttle run music vs. 20-m shuttle run |  |  | 8.3 vs. 7.9 |  |  | 177 vs.178 |  |  | 2 |
| Hébert et al., 2011^[41]^ | Handgrip |  |  |  |  |  |  | 0% |  | 1 |
| Smits-Engelsman et al., 2020^[42]^ | Standing long jump/ singe-leg stand/ dynamic balance |  |  |  |  |  |  | 0% |  | 1 |
| Ito et al., 1996^[44]^ | Trunk flexor endurance/isometric back endurance |  |  |  |  |  |  | 0% |  | 1 |
| **Total studies by item** |  | 2 | 2 | 4 | 2 | 1 | 7 | 10 | 1 |  |

DOMS: Delayed-onset soreness; HR: Heart rate. A/N: Not available data. ^$^ Questions answered by participants after the test thought Borg scale (1-10 points). ^$$^Item answered by participants 48 hours post test thought questionnaire. *After the test. **Adverse events refer to falls, sick feeling, pain and/or injury. ^#^An acceptable level of safety was considered when the items were ‘positively’ answered in at least 99 % of the cases.

**REFERENCES:**

20. Suni JH, Miilunpalo, S. I., Asikainen, T. M., Laukkanen, R. T., Oja, P., Pasanen, M. E., & Vuori, I. M. Safety and feasibility of a health-related fitness test battery for adults. Phys Ther. 1998;78(2):134-48.

28. Bruggeman BS, Vincent, H. K., Chi, X., Filipp, S. L., Mercado, R., Modave, F., & Bernier, A. Simple tests of cardiorespiratory fitness in a pediatric population. Plos one. 2020;15(9).

29. José A, & Dal Corso, S. Step tests are safe for assessing functional capacity in patients hospitalized with acute lung diseases. J Cardiopulm Rehabil Prev. 2016;36(1):56-61.

30. Borel B, Fabre, C., Saison, S., Bart, F., & Grosbois, J. M. An original field evaluation test for chronic obstructive pulmonary disease population: the six-minute stepper test. Clin Rehabil. 2010;24(1):82-93.

31. Lamoneda J, Huertas-Delgado, F. J., & Cadenas-Sanchez, C. Feasibility and concurrent validity of a cardiorespiratory fitness test based on the adaptation of the original 20 m shuttle run: The 20 m shuttle run with music. J Sports Sci. 2021;39(1):57-63.

32. Amado-Pacheco JC, Prieto-Benavides DH, Correa-Bautista JE, García-Hermoso A, Agostinis-Sobrinho C, María Alonso-Martínez A., et al. Feasibility and reliability of physical fitness tests among colombian preschool children. Int J Environ Res Public Health. 2019;16(17):3069.

34. Oja P, Laukkanen, R., Pasanen, M., Tyry, T., & Vuori, I. A 2-km walking test for assessing the cardiorespiratory fitness of healthy adults. Int J Sports Med. 1991;12(4):356-62.

35. Laukkanen RM, Oja, P., Ojala, K. H., Pasanen, M. E., & Vuori, I. M. Feasibility of a 2-km walking test for fitness assessment in a population study. Scand J Med Sci Sports. 1992;20(2):119-26.

39. España-Romero V, Artero EG, Jimenez-Pavón D, Cuenca-Garcia M, Ortega FB, Castro-Piñero J, et al. Assessing health-related fitness tests in the school setting: reliability, feasibility and safety; the ALPHA Study. Int J Sports Med. 2010;31(7):490-7.

40. Aadahl M, Zacho, M., Linneberg, A., Thuesen, B. H., & Jørgensen, T. Comparison of the Danish step test and the watt-max test for estimation of maximal oxygen uptake: the Health 2008 study. Eur J Prev Cardiol. 2013;20(6):1088-94.

41. Hébert LJ, Maltais, D. B., Lepage, C., Saulnier, J., Crête, M., & Perron, M. . Isometric muscle strength in youth assessed by hand-held dynamometry: A feasibility, reliability, and validity study: A feasibility, reliability, and validity study. Pediatr Phys Ther. 2011;23(3):289-99.

42. Smits-Engelsman B, Bonney, E., Neto, J. L. C., & Jelsma, D. L. Feasibility and content validity of the PERF-FIT test battery to assess movement skills, agility and power among children in low-resource settings. BMC Public Health. 2020;20(1):1-11.

44. Ito T, Shirado, O., Suzuki, H., Takahashi, M., Kaneda, K., & Strax, T. E. Lumbar trunk muscle endurance testing: an inexpensive alternative to a machine for evaluation. Arch Phys Med Rehabil. 1996;77(1):75-9.
